# A p53/miR-30a/ZEB2 axis controls triple negative breast cancer aggressiveness

**DOI:** 10.1038/s41418-018-0103-x

**Published:** 2018-04-17

**Authors:** Alessandra di Gennaro, Valentina Damiano, Giulia Brisotto, Michela Armellin, Tiziana Perin, Antonella Zucchetto, Michela Guardascione, Herman P. Spaink, Claudio Doglioni, B. Ewa Snaar-Jagalska, Manuela Santarosa, Roberta Maestro

**Affiliations:** 1 Oncogenetics and Functional Oncogenomics Unit, CRO Aviano National Cancer Institute, via F. Gallini 2, Aviano, 33081 PN Italy; 2 Pathology Unit, CRO Aviano National Cancer Institute, Aviano (PN), via F. Gallini 2, Aviano, 33081 PN Italy; 3 Unit of Cancer Epidemiology, CRO Aviano National Cancer Institute, Aviano (PN) via F. Gallini 2, Aviano, 33081 PN Italy; 4 Medical Oncology Unit, CRO Aviano National Cancer Institute, via F. Gallini 2, Aviano, 33081 PN Italy; 5 Molecular Cell Biology Department, Institute of Biology, Leiden University, Leiden, 2333CC The Netherlands; 6 Ateneo Vita-Salute, Department of Pathology, IRCCS Scientific Institute San Raffaele, Milan, 20132 Italy

## Abstract

Inactivation of p53 contributes significantly to the dismal prognosis of breast tumors, most notably triple-negative breast cancers (TNBCs). How the relief from p53 tumor suppressive functions results in tumor cell aggressive behavior is only partially elucidated. In an attempt to shed light on the implication of microRNAs in this context, we discovered a new signaling axis involving p53, miR-30a and ZEB2. By an in silico approach we identified miR-30a as a putative p53 target and observed that in breast tumors reduced miR-30a expression correlated with p53 inactivation, lymph node positivity and poor prognosis. We demonstrate that p53 binds the *MIR30A* promoter and induces the transcription of both miRNA strands 5p and 3p. Both miR-30a-5p and -3p showed the capacity of targeting ZEB2, a transcription factor involved in epithelial–mesenchymal transition (EMT), tumor cell migration and drug resistance. Intriguingly, we found that p53 does restrain ZEB2 expression via miR-30a. Finally, we provide evidence that the new p53/miR-30a/ZEB2 axis controls tumor cell invasion and distal spreading and impinges upon miR-200c expression. Overall, this study highlights the existence of a novel axis linking p53 to EMT via miR-30a, and adds support to the notion that miRNAs represent key elements of the complex network whereby p53 inactivation affects TNBC clinical behavior.

## Introduction

Breast cancer (BC) is the most common cancer among women. Despite significant advances in early diagnosis and treatment, metastatic spread still represents a major cause of death for BC patients. BCs are typically classified into hormone receptor positive (HR; estrogen receptor and/or progesterone receptor), HER2/ERBB2/NEU-positive or triple-negative tumors (TNBC, negative for hormonal and HER2 receptors) according to their receptor status, as assessed by immunohistochemistry. In 2000, Perou et al. [1] suggested a molecular classification of BC into four major subgroups based on the transcriptional profile. These four molecular BC subtypes overlap only in part with the conventional receptor classification: luminal A and luminal B, including most of HR-positive tumors; HER2-positive tumors; and basal-like BC, grossly corresponding to TNBC [1]. Among the different BC subtypes, TNBC/basal-like tumors feature a particularly aggressive behavior: compared to the other BC subtypes, TNBC patients tend to relapse earlier and have higher recurrence rates in the first years after diagnosis [2]. In fact, in the absence of an approved target therapy for TNBC, radiotherapy and chemotherapy still represent the mainstay of treatment [3]. Unfortunately, primary or secondary resistance often occurs, which contributes to the dismal prognosis of these tumors [3].

The inactivation of the tumor suppressor p53 is thought to play a major role in the aggressiveness of TNBC by promoting metastatic spreading, resistance to therapy and relapse [4]. In TNBC/basal-like BC, *TP53* alterations involve over 80% of the tumors and are mostly represented by disrupting mutations (gene deletions or insertions). Instead, only 19% of HR-positive/luminal tumors present *TP53* alterations (12% of luminal A, 29% of luminal B) that are primarily missense mutations [5]. These facts support the notion that p53 contributes to TNBC/basal-like BC mostly through loss of tumor suppressive functions, rather than through gain of oncogenic activities (gain-of-function p53 mutations).

Loss of function of p53 results in the abolition of p53-mediated checkpoints and stress responses, and recent evidence points to a role of microRNAs (miRNAs) in these contexts [6–8]. miRNAs are small, non-coding RNAs that, through base pairing with target messenger RNA (mRNA) molecules, regulate gene expression by inducing either mRNA degradation or inhibition of translation [9, 10]. p53 has been described to regulate the expression of a number of miRNAs that mediate p53 control over several biological processes including cell cycle, epithelial–mesenchymal transition (EMT) and cell plasticity, survival and metabolism [6,11–14]. On these grounds we sought to investigate in deeper detail the contribution of miRNAs as mediators of p53 tumor suppressive functions in the context of TNBC/basal-like tumors.

## Results

### miR-30a is downregulated in TP53-inactivated TNBC and correlates with poor outcome

To investigate the possible contribution of a p53/miRNA pathway in the pathogenesis and aggressive behavior of BC, we took advantage of the in silico predictor of p53-responsive elements developed by Gowrisankar and Jegga [15]. The algorithm identified 23 miRNAs as high confidence p53 targets (score ≥ 3). The interrogation of the publicly available TCGA (The Cancer Genome Atlas) BC dataset (at http://tcga-data.nci.nih.gov/tcga/findArchives.htm [16]) highlighted 13/23 miRNAs as significantly modulated in *TP53*-mutated (including missense mutations, deletions and insertions) compared to *TP53* wild-type BC (Table 1). Among these, miR-30a stood out as it was the only miRNA to be significantly downregulated in *TP53*-mutated tumors. The presence of several putative p53-responsive elements in the promoter region of miR-30a was also confirmed by the MatInspector tool [17]. This finding was suggestive of a potential control of p53 over miR-30a gene expression.

**Table 1:** Differentially expressed miRNAs predicted to be p53-regulated in *TP53*-mutated and *TP53* wild-type breast cancers

miRNA predicted to be regulated by p53^a^	Transactivation score^a^	LOG2 fold change^b^	*P*-value^c^
miR-146a	3	1.2	2.1E−07
let-7i	3	0.5	1.6E−06
miR-671	4	0.6	1.9E−06
**miR-30a**	**3**	**-0.8**	**8.5E−06**
miR-138-2	3	3.1	1.9E−04
miR-138-1	4	3.7	3.6E−04
miR-15b	3	0.8	4.8E−04
miR-615	3	0.8	5.6E−04
miR-9-2	3	2.4	2.0E−03
miR-196a-2	3	0.6	9.2E−03
miR-181a-1	3	0.3	1.0E−02
miR-191	4	0.4	1.3E−02
miR-328	4	0.3	3.4E−02
miR-490	3	2.1	9.4E−02
miR-194-1	3	0.2	1.1E−01
miR-302b	4	ND	2.3E−01
miR-34a	3	0.2	2.4E−01
miR-135a-2	3	–1.6	2.6E−01
miR-153-2	3	–0.3	3.9E−01
miR-1-1	3	–3.0	4.5E−01
miR-124-3	4	1.2	5.0E−01
miR-100	3	0.1	7.2E−01
miR-29b-2	3	0.0	8.3E−01

^a^Transactivation score as calculated by Gowrisankar and Jegga [15]

^b^Data are reported as LOG2 fold change between TP53-mutated (82 cases) and wild-type (163 cases) breast cancers (TCGA series; strands from the same miRNA were jointly analyzed)

^c^The top 13 miRNAs were differentially expressed (*p* < 0.05) in TP53-mutated vs wild-type breast cancers

**Fig. 1:**
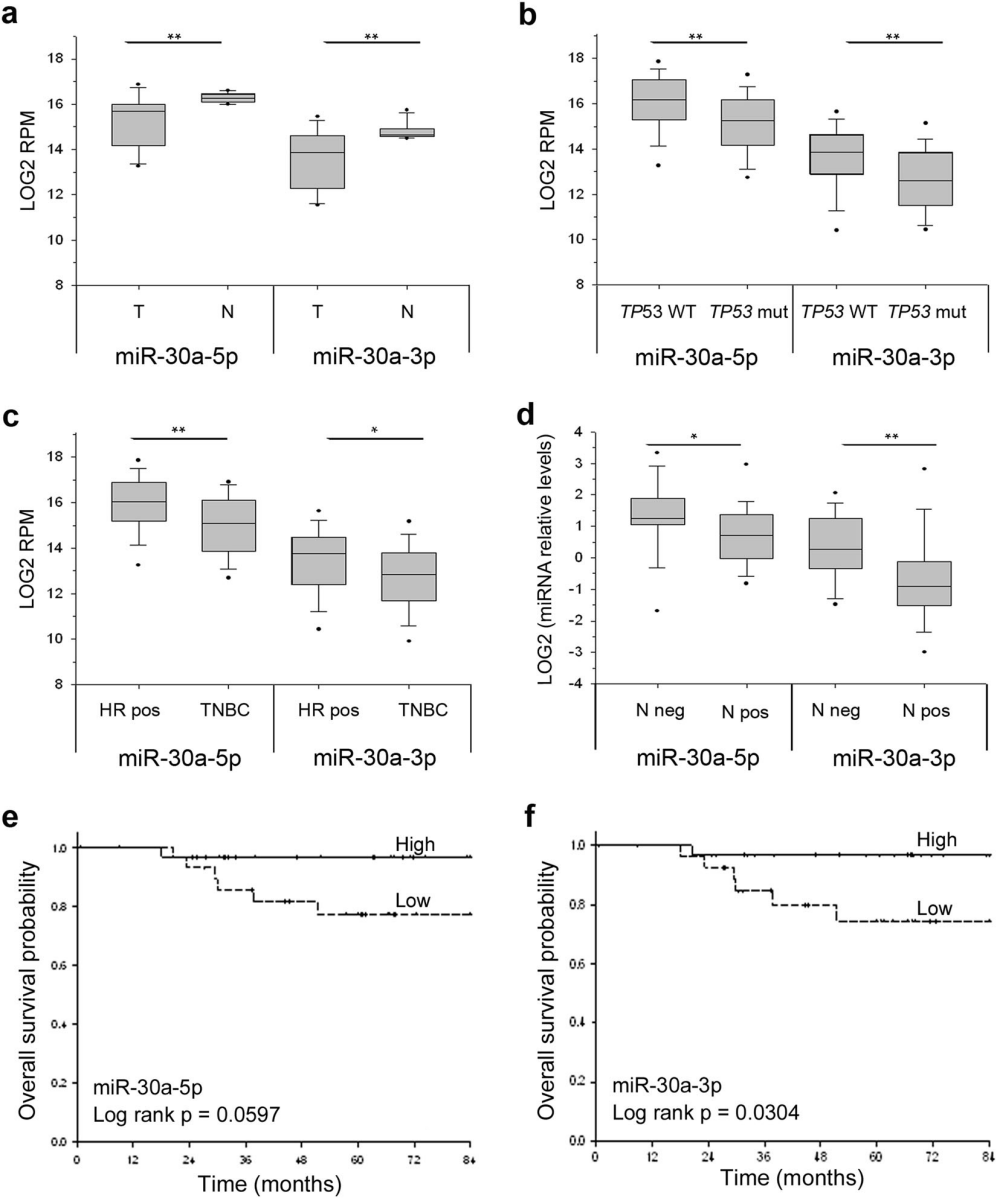
miR-30a-5p and miR-30a-3p expression in breast cancers of the TCGA series (**a**–**c**) and of the in-house series (**d–f**). TCGA series: **a** the expression levels of miR-30a-5p and miR-30a-3p (expressed as LOG2 RPM) are lower in breast tumors (T) compared to matched normal breast tissues (N; 12 cases). **b**, **c** miR-30a-5p and miR-30a-3p levels are lower in the 82 BCs carrying *TP53* mutations (missense, nonsense, frameshift; *TP53* mut) compared to the 163 *TP53* wild-type tumors (*TP53* WT) (**b**) and in TNBC (27 cases) compared to hormone receptor-positive tumors (HR, 204 cases) (**c**). The *p* values were calculated by Mann–Whitney rank sum test; *p < 0.05, **p < 0.01. In-house series: **d** low levels of either miRNA strand are associated with lymph node positivity. Data are reported as LOG2 of miRNA relative levels; *p* values were calculated by two-tailed *t*-test; *p < 0.05, **p < 0.01. **e**, **f** Kaplan–Meier analysis showing overall survival in the TNBC patients classified according to miR-30a-5p (**e**) and miR-30a-3p (**f**) median levels. Survival curves are truncated at 84 months. In (**a**–**d**) lines within the boxes mark the median, boundaries represent the 25th and the 75th percentiles and whiskers below and above the boxes indicate the 5th and 95th percentiles

To address the actual existence of a p53/miR-30a interplay in the context of BC we further interrogated the TCGA database for miR-30a expression. In the biogenesis of a miRNA, the primary miRNA (pri-miRNA) transcribed from the miRNA gene is first processed into a precursor miRNA (pre-miRNA) to then form a guide miRNA, which regulates target mRNA expression [9]. The other strand, named passenger strand, is usually degraded, but there are examples in which both strands give rise to mature miRNAs [9, 10]. Analysis of miR-30a expression revealed that this was indeed the case for miR-30a. In fact, both miR-30a-5p and miR-30a-3p were expressed in normal breast tissues, and both were significantly downregulated in BC (*p* < 0.01, Fig. 1a), with a high degree of reciprocal correlation (*r* = 0.80, *p* < 0.01). Interestingly, the expression of both miR-30a strands was significantly lower in *TP53-*inactivated BCs compared to *TP53* wild-type BCs, irrespective of the type of mutation (Fig. 1b; Supplementary Figure S1a). Moreover, miR-30a downregulation was more dramatic in TNBC compared to HR-positive tumors (Fig. 1c). The difference in miR-30a expression observed between TNBC and HR-positive tumors was most likely attributable to p53, as the statistical difference between the two subtypes was lost when corrected for *TP53* gene status (Supplementary Figure S1b).

To investigate a possible role of miR-30a in the aggressive behavior of TNBC, we then interrogated an in-house cohort of 59 consecutive TNBCs with full clinical history (Table 2): the expression of miR-30a, particularly miR-30a-3p, inversely correlated with lymph node positivity (Fig. 1d), shorter disease-free (data not shown) and overall survival (Fig. 1e, f). Taken together, these results suggested a concerted action of p53 and miR-30a in the control of TNBC clinical outcome.

**Table 2:** Triple-negative breast cancers cases: distribution according to miR-30a (5p and 3p) expression, demographics and clinical pathological features

		miR-30a-5p			miR-30a-3p		
	Total (*N*=59)	Low^a^ (*N*=29)	High^a^ (*N*=30)	*P*-value	Low^a^ (*N*=29)	High^a^ (*N*=30)	*P*-value
Median follow-up (months)	63.2	61.0	65.4	0.87^c^	51.3	67.7	0.06^c^
Median age at diagnosis (yrs)	51	49	52	0.50^c^	52	50	0.13^c^
	No. (%)	No. (%)	No. (%)		No. (%)	No. (%)	
Tumor size^b^							
T1	28 (52.8)	12 (42.9)	16 (64.0)	0.09^d^	11 (39.3)	17 (68.0)	0.06^d^
T2	22 (41.5)	13 (46.4)	9 (36.0)		14 (50.0)	8 (32.0)	
T3–T4	3 (5.7)	3 (10.7)	0 (0.0)		3 (10.7)	0 (0.0)	
Lymph nodes^b^							
N0	28 (47.5)	10 (35.7)	18 (75.0)	0.01^d^	9 (32.1)	19 (79.2)	<0.001^d^
N+	24 (40.7)	18 (64.3)	6 (25.0)		19 (67.9)	5 (20.8)	
Metastasis							
M0	54 (91.5)	28 (96.6)	26 (86.7)	0.35^d^	27 (93.1)	27 (90.0)	1.00^d^
M+	5 (8.5)	1 (3.5)	4 (13.3)		2 (6.9)	4 (10.0)	
TNM stage							
I	19 (32.2)	8 (27.6)	11 (36.7)	0.80^d^	7 (24.1)	12 (40.0)	0.43^d^
II	25 (42.4)	13 (44.8)	12 (40.0)		13 (44.8)	12 (40.0)	
III–IV	15 (25.4)	8 (27.6)	7 (23.3)		9 (31.0)	6 (20.0)	
Tumor grade^b^							
G1–G2	3 (5.1)	1 (3.6)	2 (6.7)	1.00^d^	0 (0.0)	3 (10.4)	0.24^d^
G3	55 (93.2)	27 (96.4)	28 (93.3)		29 (100.0)	26 (89.7)	
Radiation treatment^b^							
No	23 (45.1)	10 (38.5)	13 (52.0)	0.40^d^	11 (45.8)	12 (55.6)	1.00^d^
Yes	28 (54.9)	16 (61.5)	12 (48.0)		13 (54.2)	15 (44.4)	
Pharmacological treatment^b^							
No	9 (16.1)	3 (11.1)	6 (20.7)	0.47^d^	4 (14.8)	5 (17.2)	1.00^d^
Yes	47 (83.9)	24 (88.9)	23 (79.3)		23 (85.2)	24 (82.8)	
Drugs^b^							
Anthracycline	21 (47.7)	14 (60.9)	7 (33.3)	0.23^d^	11 (52.4)	10 (43.5)	0.73^d^
Anthracycline/Taxanes	11 (25.0)	4 (17.4)	7 (33.3)		4 (19.0)	7 (30.4)	
CMF	12 (27.3)	5 (21.7)	7 (33.3)		6 (28.6)	6 (26.1)	

*CMF* cyclophosphamide, methotrexate and 5-fluorouracil

^a^The median value was used as cut-off

^b^The sum does not add up to the total because of some missing values

^c^Mann–Whitney–Wilcoxon test

^d^Fisher’s exact test

### miR-30a is a direct transcriptional target of p53

**Fig. 2:**
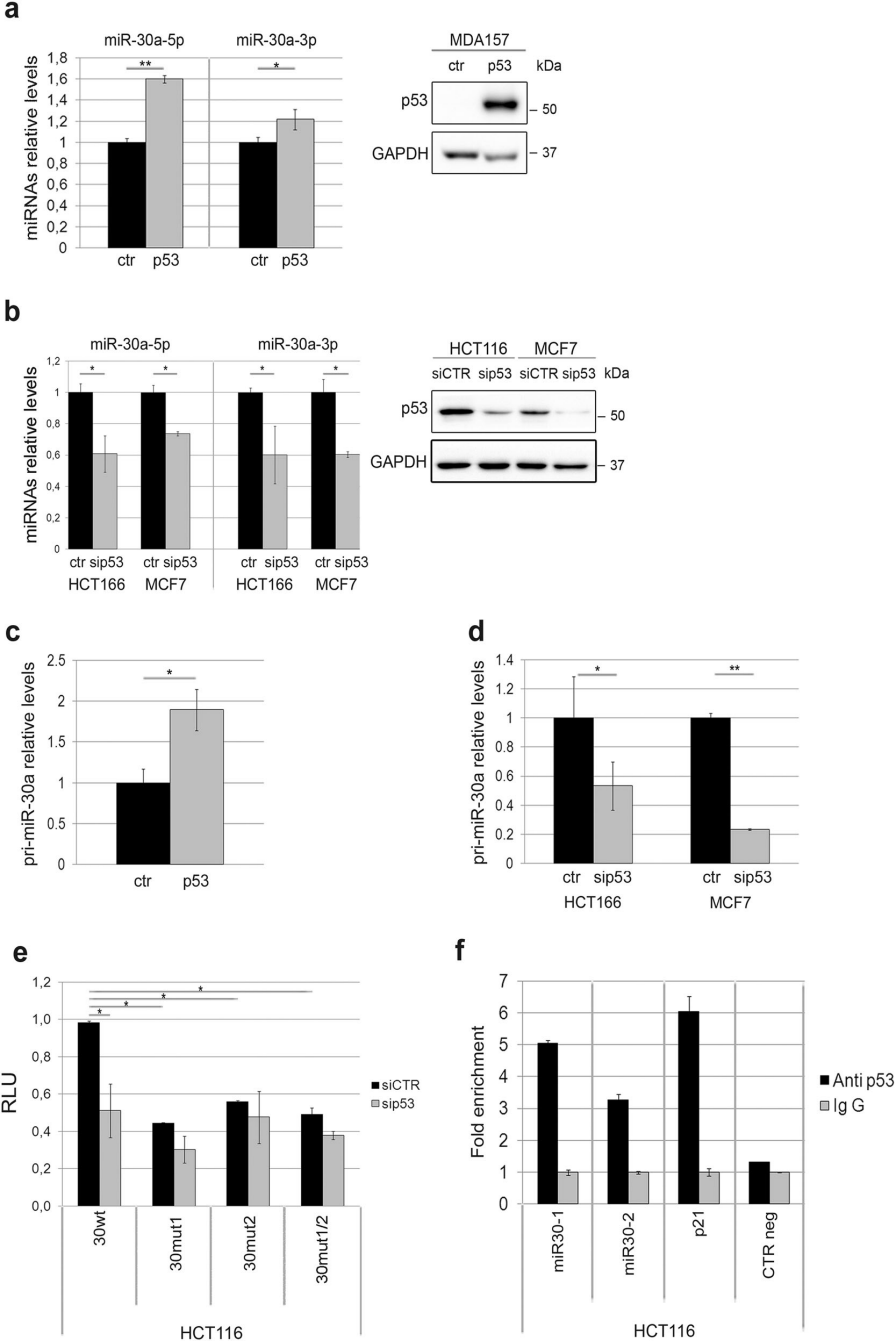
p53 regulates miR-30a-5p and miR-30a-3p expression. **a** Ectopic p53 expression in MDA157 results in an augment of mature miR-30a-5p and miR-30a-3p levels. The immunoblot on the right shows p53 expression in MDA157 engineered cells. GAPDH was used as a loading control. **b** p53 silencing in HCT116 results in a decrement of both miR-30a-5p and miR-30a-3p. The extent of p53 silencing is shown in the right panel. **c** Ectopic p53 induces an increase of pri-miR-30a levels in MDA157. **d** Decrease of pri-miR-30a levels in p53-depleted HCT116 and MCF7 cells. **a**–**d** Gray columns represent p53-modulated samples; black columns represent control cells. **e** p53 regulates the *MIR30A* promoter. Silencing of p53 results in a decrement of the *MIR30A* promoter activity (30wt). The mutagenesis of the two p53 binding sites, singularly (30mut1, 30mut2) or in combination (30mut1/2), abrogates this effect. Results represent the mean value of three independent experiments ± SD. **f** p53 binds the miR-30a promoter. Chromatin immunoprecipitation was performed with the DO-1 anti-p53 monoclonal antibody on HCT116 genomic DNA. Isotype-matched pre-immune mouse IgG was used as a negative control. The immunoprecipitated chromatin was assayed for the enrichment of the target *MIR30A* promoter (miR30-1 and 2, the regions encompassing the two p53BS) by qPCR. The p53 binding region of the p21 promoter and an irrelevant genomic region (CTR neg) [59] were used as positive and negative control, respectively. Data are reported as fold enrichment over control samples (immunoprecipitation with pre-immune IgG) p values were calculated by two-tailed t-test; *p < 0.05, **p < 0.01.

Based on the data accumulated, we sought to investigate in detail the p53/miR-30a interplay. In keeping with the in silico predictions of a p53-mediated control of miR-30a, we observed that modulation of p53 expression in tumor cell lines affected miR-30a transcription. Specifically, ectopic expression of p53 in *TP53*-null cells (MDA157) or small interfering RNA (siRNA)-mediated downregulation of p53 in *TP53* wild-type cells (HCT116 and MCF7) associated with a concordant variation in the expression of both miR-30a strands (Fig. 2a, b). The finding that modulation of p53 affected miR-30a expression at the level of primary transcript (pri-miRNA; Fig. 2c, d) further supported the notion of a control of p53 over miR-30a transcription.

To validate this hypothesis we generated a *MIR30A* reporter plasmid in which the *MIR30A* promoter, either wild type or mutagenized in the two p53 binding sites with the highest prediction score according to MatInspector (p53BS, miR30-1 and miR30-2), was cloned upstream of the luciferase gene (Supplementary Figure S2a). Both p53BS single site (30mut1 and 30mut2) and p53BS dual site mutant reporters (30mut1/2 with both the predicted p53BS mutagenized) were generated. Reporter assays indicated that silencing of p53 (Supplementary Figure S2b) affected the activity of the *MIR30A* wild-type reporter, while it had negligible effects on the reporters in which either one or both p53BS were destroyed (Fig. 2e comparison black vs gray matched columns). Moreover, in p53 proficient cells the luciferase activity was significantly reduced in p53BS mutated reporters compared to the wild-type one (Fig. 2e comparison between black columns); this difference was erased after silencing of p53 (Fig. 2e comparison between gray columns). Overall these data added support to the notion that miR-30a is a direct transcriptional target of p53.

To ultimately demonstrate that p53 actually sits on the *MIR30A* promoter, we performed chromatin immunoprecipitation (ChIP) experiments. HCT116 cell lysates were immunoprecipitated using a p53-specific antibody and the regions encompassing the two putative p53BS elements on the *MIR30A* promoter (miR30-1 and miR30-2) were amplified and quantified by quantitative PCR (qPCR). Pre-immune IgG isotype antibodies were used in a mock immunoprecipitation as a negative control/background signal; the p53 binding region of the p21 promoter was instead used as a positive control in PCR reactions. Quantitative PCR-ChIP confirmed a strong enrichment of the amplicons encompassing the two p53BS in anti-p53 ChIP compared to the background (control IgG-ChIP, Fig. 2f). The identification of functional binding sites for p53 in the *MIR30A* promoter region compellingly demonstrated that miR-30a is a direct transcriptional target of p53.

### miR-30a-5p and miR-30a-3p target ZEB2

**Fig. 3:**
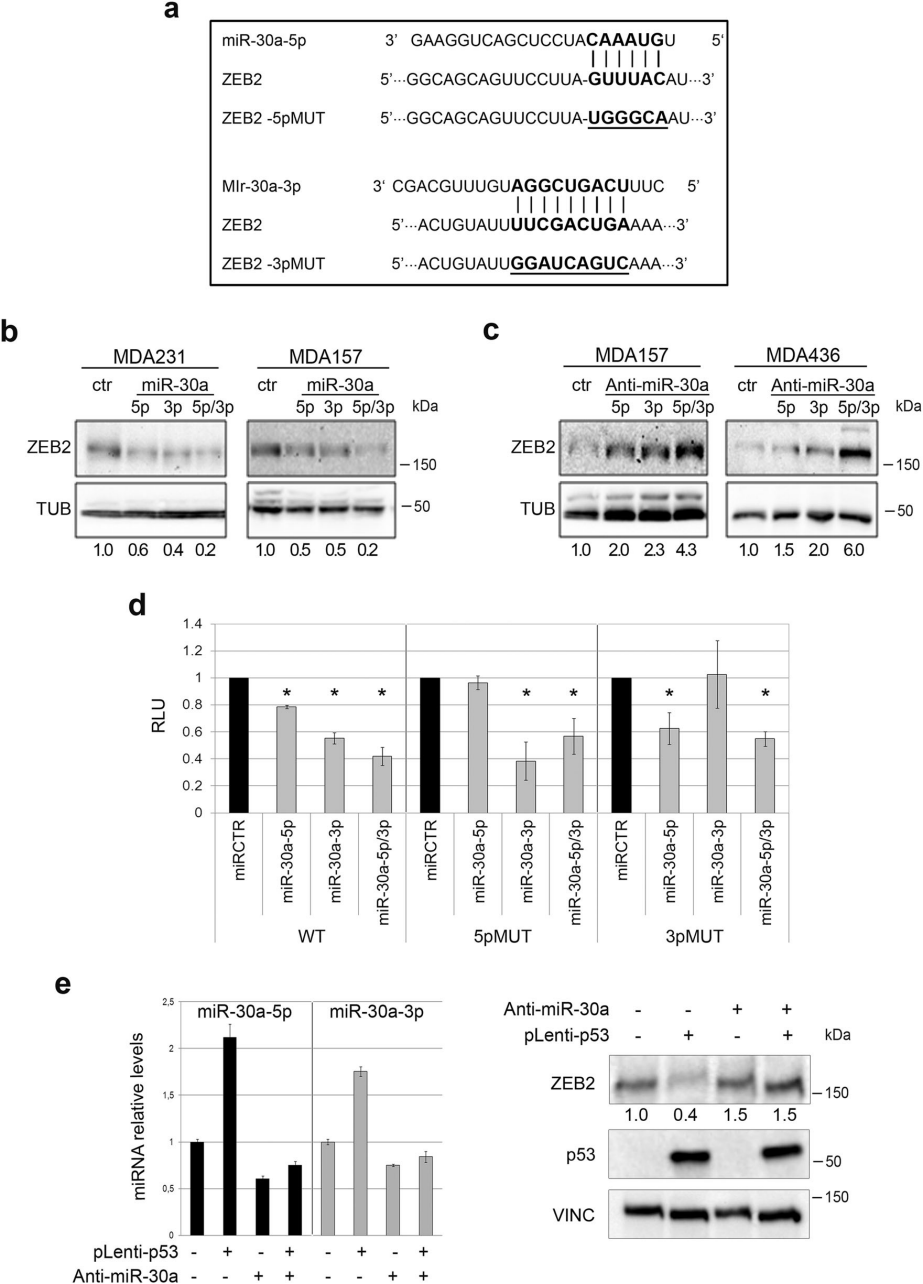
miR-30a-5p and miR-30a-3p inhibit ZEB2 and mediate the p53 control over ZEB2. **a** Alignment of *ZEB2* 3’UTR with miR-30a-5p and miR-30a-3p (Targetscan). Nucleotides mutagenized to disrupt the miRNA/mRNA base pairing are underlined. **b** Immunoblots showing the modulation of ZEB2 in response to miR-30a (5p, 3p or both) ectopic expression and **c** after anti-miR-mediated inhibition of miR-30a. ZEB2 relative levels, normalized over Tubulin (loading control), are reported below. **d** miR-30a targets the 3′UTR of ZEB2: luciferase activity of the *ZEB2* 3’UTR reporter, wild-type (WT) or mutated in the miR-30a-5p and -3p binding sites (5pMUT and 3pMUT), was measured 48 h post transfection of MDA231 cells with the indicated miRNAs. miRCTR was set as a reference. CMV-*Renilla* was used for normalization. Results represent the mean value ± SD of three experiments. The asterisks (*) indicate the comparisons of miR-30a vs miRCTR that are statistically significant (*p* < 0.05). **e** miR-30a-5p and miR-30a-3p expression in MDA157 stably transduced with p53 (pLenti-p53+) or pLenti-GFP (pLenti-p53−), in the absence (anti-miR-30a−) or presence (anti-miR-30a+) of an anti-miRNA targeting miR-30a. Immunoblots for ZEB2, p53 and Vinculin (loading control) are shown on the right. Numbers below the blot indicate ZEB2 relative levels normalized over Vinculin

Having collected evidence that indicates that p53 directly controls miR-30a and that p53 inactivation results in miR-30a downregulation, we then sought to deepen how this interplay impinged upon the aggressive behavior of TNBC. To gain insights on the pathways regulated by the p53/miR-30a axis, we interrogated several in silico prediction tools (microRNA Data Integration Portal-mirDIP [18]). Computational analyses identified several miR-30a targets, among which include SNAI1, SNAI2 and ZEB2. miR-30a is known to participate to the control of EMT and cell plasticity/stemness and we confirmed in our cell models the ability of miR-30a to impinge upon these phenotypes (Supplementary Figure S3a-d). SNAI1 and SNAI2 have been previously reported to be regulated by miR-30a [19–21]. Instead, the ability of miR-30a to target ZEB2 was a new finding (Fig. 3a). Intriguingly, both miR-30a strands were predicted to bind ZEB2 mRNA.

ZEB2 is a transcription factor that plays a crucial role in the control of cell plasticity and in the orchestration of the EMT phenomena that occur during embryogenesis [22]. In analogy with its embryonic function, ZEB2 overexpression has also been shown to promote EMT in tumors, including BC [23–25]. Accordingly, ZEB2 sustained the cell motility of TNBC cell models. In fact, ZEB2 downregulation significantly reduced in vitro migration of MDA231, MDA157, BT549, Hs578T and HCC1395 TNBC cell lines (Supplementary Figure S4a). Intriguingly, even a reduction of expression to 50% sufficed to impact on cell motility.

Since ZEB2 is also expressed by stromal cells [26], to specifically address the involvement of ZEB2 in human tumor cells we sought to use an in situ approach. Immunohistochemical staining revealed that BCs, particularly the TNBC/basal-like subset, did express the ZEB2 protein (Supplementary Figure S4b and c).

Based on these findings, we explored in vitro whether miR-30a affected ZEB2 expression. Modulation of miR-30a expression in BC cell lines inversely correlated with ZEB2 levels: ectopic expression of miR-30a (5p, 3p or both strands, 5p/3p) resulted in ZEB2 decrease (Fig. 3b and Supplementary Figure S5a); conversely, anti-miR-mediated inhibition of miR-30a elicited an increase in ZEB2 levels (Fig. 3c and Supplementary Figure S5b).

To ascertain whether ZEB2 was a direct target of miR-30a-5p and miR-30a-3p, reporter assays were performed using luciferase reporter constructs carrying the 3’ untranslated region (UTR) sequence of ZEB2, either wild type (WT) or mutated in the seeds for the two miR-30a strands (5pMUT, 3pMUT; Fig. 3a). Transfection of miR-30a-5p and miR-30a-3p significantly inhibited the expression of the ZEB2 3’UTR wild-type reporter (WT), while it had no effect on the reporter in which the cognate miR-30a seeds were mutagenized. The combination of the two miRNAs showed a quasi-additive effect (5pMUT, 3pMUT; Fig. 3d and Supplementary Figure S5c).

Overall, these data indicated that miR-30a (5p and 3p strands) exerts an epigenetic control over ZEB2 mRNA. To address whether p53 entered into the miR-30a/ZEB2 equation, we ectopically expressed p53 in MDA157. This cell line was selected because it is p53 null and because of its negligible expression of miR-200c [27], a possible confounding factor. In fact, miR-200c is a p53-regulated miRNA previously reported to be connected to ZEB2 via reciprocal feedback loop [13, 28]. The augment of miR-30a induced by ectopic p53 was paralleled by a reduction in ZEB2 protein levels (Fig. 3e). The existence of a p53 control on ZEB2 via miR-30a was confirmed by the finding that anti-miR-mediated interference of miR-30a abrogated the ability of p53 to affect ZEB2 (Fig. 3e, Supplementary Figure S6a-d). Taken together, these data indicate that, beside the previously reported p53-miR-34a-SNAI1 axis [11, 14], p53 exerts a control over EMT also through the new route involving miR-30a and ZEB2.

### The miR-30a/ZEB2 axis controls TNBC tumor spreading

**Fig. 4:**
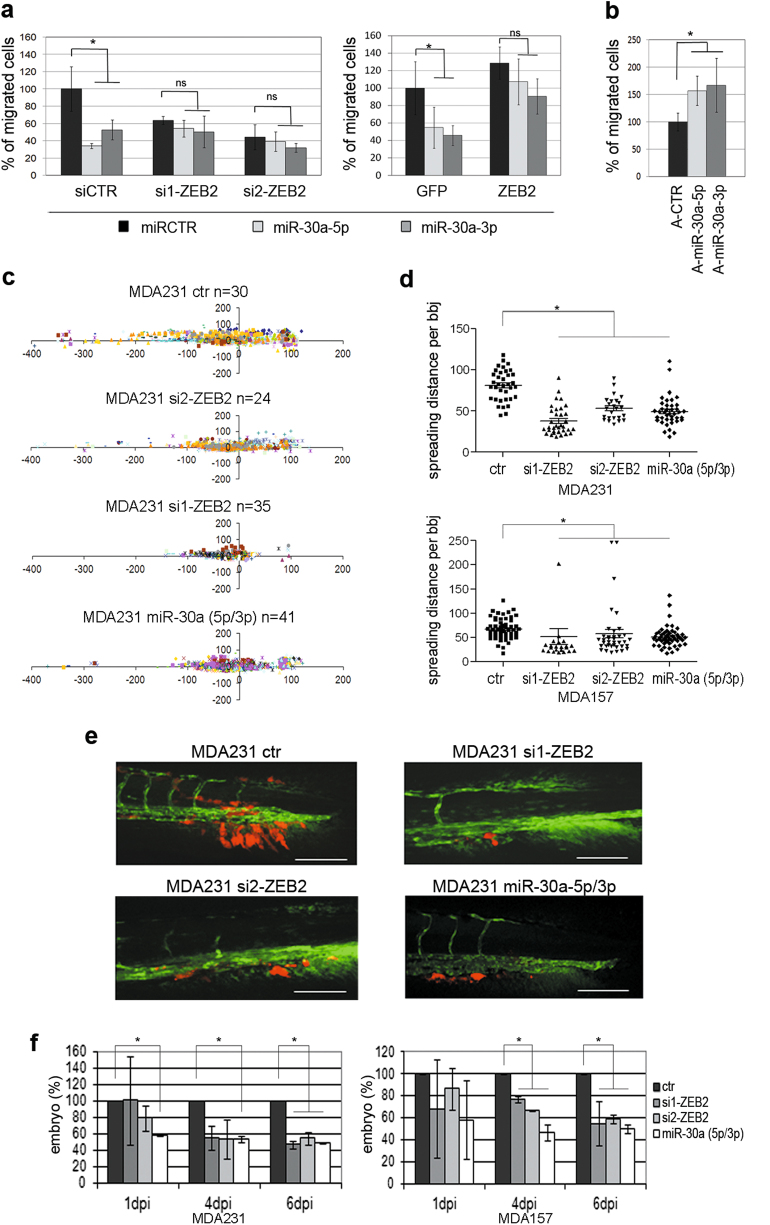
miR-30a (5p/3p) overexpression results in a reduced BC cell migration in vitro and in a zebrafish xeno-transplantation model. **a** In vitro cell migration assay of MDA231 engineered to ectopically express miR-30a-5p, miR-30a-3p or control miRNA (miRCTR) in a ZEB2-proficient (siCTR) or ZEB2-deficient (si1-ZEB2 and si2-ZEB2) context (left panel). The right panel shows the migration capacity of MDA231 engineered to stably overexpress ZEB2 or GFP, used as a negative control. **b** In vitro cell migration assay of MDA231 transfected with anti-miR-30a-5p (A-miR-30a-5p), anti-miR-30a-5p (A-miR-30a-5p) or control anti-miRNA (A-CTR). The percentage of transmigrated cells was measured at 7 h post seeding. Data represent the mean of three independent experiments; *p < 0.05. **c** Scatter plot representing cell dissemination in zebrafish embryos of mCHERRY MDA231 transiently silenced for *ZEB2* (si1-ZEB2 and si2-ZEB2) or engineered to stably express miR-30a (5p/3p). Cells transfected with an empty vector were used as a control (ctr). Cells were implanted in the yolk sac of 2-day-old embryos (fli1:EGFP strain). Embryos were automatically imaged at 6 dpi. Dots represent single cells; colors identify each microinjected embryo; *x*-axis indicates the migration from the injection point (0,0) toward the head (positive values) or the tail (negative values); *n* indicates the number of embryos analyzed. **d** Spreading distance of MDA231 and MDA157 calculated from data represented in (**c**) and in Supplementary Figure S8b, respectively; *p < 0.05. **e** Representative images of zebrafishes injected with MDA231 cells at 6 dpi. Cells were injected into the blood circulation (duct of Cuvier) of 2-day-old zebrafish embryos. Scale bar = 100 µm. **f** Percentages of embryos that show caudal micrometastatic colonization after injection with MDA231 or MDA157 cells engineered with control vector (ctr), miR-30a (5p/3p) or silenced for *ZEB2* (si1-ZEB2 and si2-ZEB2). The percentage of ctr embryos showing metastasis was arbitrarily set to 100. Data are shown at 1, 4 and 6 dpi. Figures represent the results of two independent experiments

To address the biological implication of the p53/miR-30a/ZEB2 axis in TNBC biology, we ascertained whether miR-30a actually affected tumor spreading via ZEB2. To this end, we ectopically expressed miR-30a-5p, miR-30a-3p or the control miRNA (miRCTR) in MDA231 cells silenced (siRNA) for ZEB2 or in MDA231 engineered to overexpress ZEB2. Either miR-30a strand inhibited migration of ZEB2-proficient but not ZEB2-deficient MDA231 cells. Moreover, ZEB2 overexpression counteracted the inhibitory effect of miR30a-5p and -3p (Fig. 4a and Supplementary Figure S7a-d).

In addition, anti-miR-mediated block of miR-30a affected cell motility (Fig. 4b). Anti-miR-30a-5p and anti-miR-30a-3p induced an increase in cell migration of comparable extent. Noteworthy, both anti-miRNAs elicited ZEB2 upregulation. In contrast, SNAI1, which is reported to be targeted by miR-30a-5p [20, 21], was upregulated only in response to the cognate anti-miR-30a-5p (Supplementary Figure S7e-f). Thus, the modulation in cell motility induced by anti-miR-30a-3p in this experimental system correlated only with ZEB2 upregulation. This supports a specific role for ZEB2 in the control exerted by miR-30a over cell motility.

To validate these concepts in vivo, we performed xenotransplant experiments using the zebrafish embryo as a model, whose transparency enables direct visualization of fluorophore-labeled tumor cells [29, 30]. MDA231 and MDA157 cells, either silenced for *ZEB2* (si1-ZEB2, si2-ZEB2) or ectopically expressing miR-30a (5p/3p), were injected in the yolk sac of zebrafish embryos to monitor tumor cell spreading and in the cardinal vein (duct of Cuvier) to monitor tail fin invasion [31]. Both ZEB2 silencing and miR-30a induction resulted in reduced tumor cell migration (Fig. 4c, d and Supplementary Figure S8a-b) and halved tail fin invasion (Fig. 4e, f and Supplementary Figure S8c), indicating a diminished tumor cell extravasation and distal dissemination.

Overall, these results corroborate the concept that the miR-30a/ZEB2 axis, which is under the control of p53, is involved in tumor cell invasion and distal spreading.

### The p53/miR-30a/ZEB2 axis impinges upon miR-200c expression

Finally, in the light of the previously reported negative control of ZEB1 and ZEB2 over miR-200 [28, 32, 33], we predicted that miR-30a, by repressing ZEB2, would in turn affect miR-200c.

**Fig. 5:**
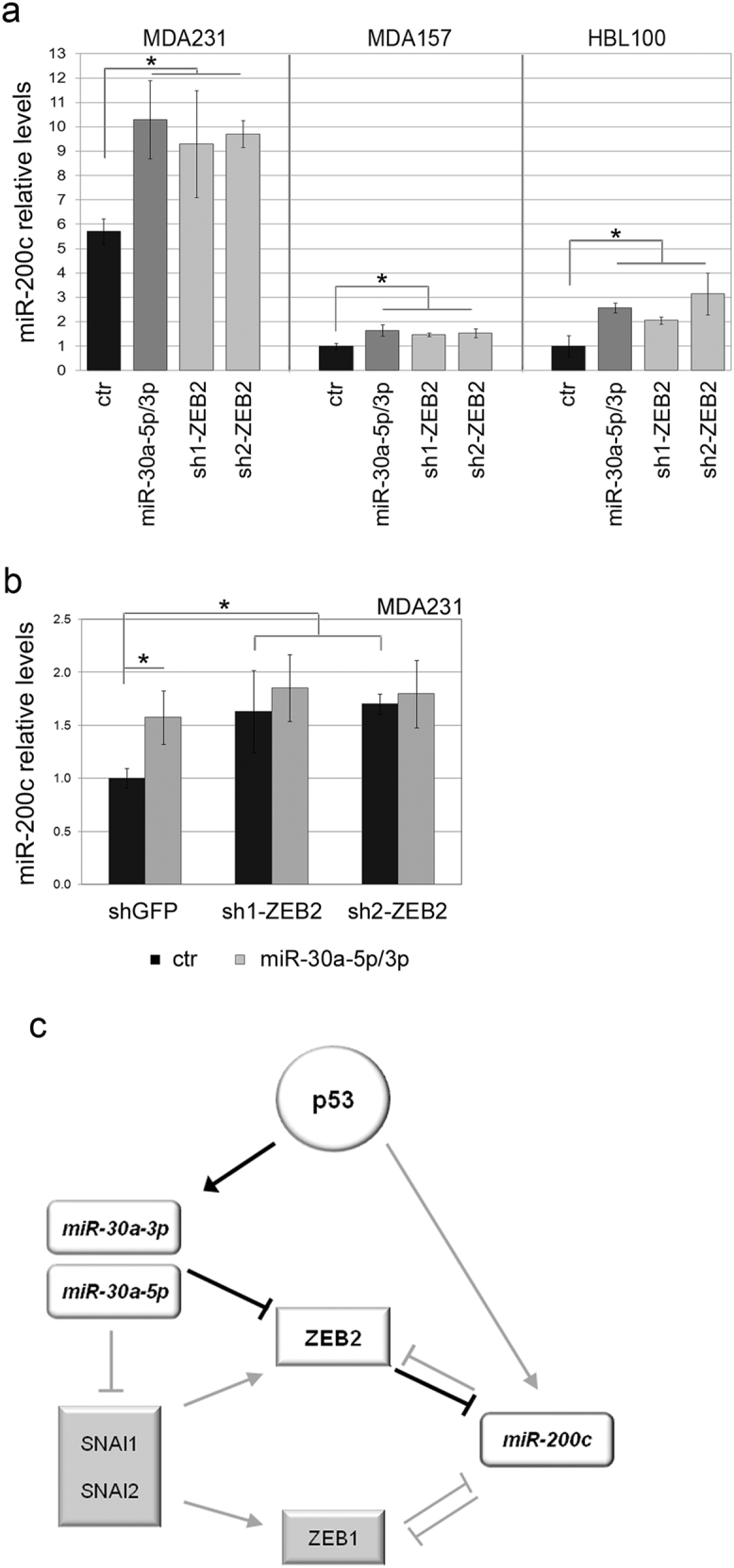
The p53/miR-30a/ZEB2 axis impinges upon miR-200c. **a** miR-200c levels in MDA231, MDA157 and HBL100 cell lines engineered to express miR-30a (5p/3p) or silenced for ZEB2 (sh1-ZEB2; sh2-ZEB2). Control vectors (pLenti6GFP and shGFP) yielded similar values and are here represented once as ctr. miR-200c levels in HBL100 ctr were set to 1. **b** miR-200c levels in MDA231 proficient (shGFP) or deficient (sh1-ZEB2, sh2-ZEB2) for ZEB2 expression, in the absence (ctr, pLenti6GFP) or presence (miR-30a-5p/3p) of ectopic miR-30a; *p < 0.05. **c** A unifying model of the new p53/miR-30a/ZEB2 axis (highlighted in bold) involved in TNBC

In accord with this hypothesis, ectopic expression of miR-30a (5p/3p) resulted in an upregulation of miR-200c that was paralleled by a marked ZEB2 downregulation (Fig. 5a, Supplementary Figure S9a and b). Instead, no major and reproducible variations in ZEB1 were observed. These results support a role for the miR-30a/ZEB2 axis in the control of miR-200c. Accordingly, silencing of ZEB2, which yielded an augment of miR-200c of similar extent to that induced by miR-30a, nullified the ability of this miRNA to induce miR-200c (Fig. 5a, b, Supplementary Figure S10a and b). The interplay between the miR-30a/ZEB2 axis and miR-200c in BC (Fig. 5c) was somehow corroborated by the finding that the miR-30a was positively correlated to miR-200c in both the TCGA and in the in-house TNBC series (Supplementary Figure S11a-d).

## Discussion

Several lines of evidence demonstrate that inactivation of p53, the tumor suppressor most frequently involved in TNBC, affects phenotypes such as EMT and cell plasticity/stemness that contribute to tumor progression and poor response to therapies [5, 7, 34–37]. As a transcription factor, p53 controls the expression of several genes involved in these phenotypes, including miRNAs [6, 13, 14].

In an attempt to elucidate the mechanisms whereby p53 inactivation contributes to the aggressive behavior of BC via miRNA, we identified a novel axis involving p53, miR-30a and ZEB2. In particular, we found that the expression of miR-30a (both miR-30a-5p and miR-30a-3p) was significantly reduced in human BC samples carrying *TP53* gene alterations, primarily TNBC, and inversely correlated with patients’ survival. This phenomenon was attributable to loss of wild-type p53 activity, rather than to gain of oncogenic functions [38], as miR-30a downregulation was observed not only in tumors with missense p53 mutations encoding a dysfunctional full-length protein, but also in tumors carrying alterations resulting in p53 protein loss.

The miR-30 family, which includes five members (miR-30a, -30b, -30c, -30d, -30e), has been implicated in the pathogenesis of different tumor types [39]. Although they share the same seed sequence, the various members differ for compensatory sequences which account for their specificity and for their diverse and sometime opposite roles in the regulation of cell proliferation, EMT and apoptosis [19–21, 40–45]. As about miR-30a, loss of miR-30a-5p has been reported to favor tumor dissemination and chemoresistance by promoting EMT [19, 20, 40, 42, 43, 46]. We propose that miR-30a mediates the control of p53 over these phenotypes, thus contributing to the aggressive behavior of p53-inactivated breast cancers. Specifically, we show that miR-30a is a direct p53 transcriptional target that is downregulated in response to p53 inactivation. Intriguingly, a study conducted on a melanoma cell line has recently suggested that p53 may repress miR-30a by binding the MIR30A promoter to a more proximal region than the one here described [47]. Although we cannot exclude p53 may exert on miR-30a different effects depending on the cell context, our data compellingly demonstrate that there is a positive functional interplay between p53 and miR-30a, both in human BC and in in vitro cell models. In keeping with our findings, miR-30a was among the miRNAs positively modulated in *TP53* wild-type vs *TP53*-null HCT116 colon cancer cells [48].

Moreover, we provide evidence that ZEB2 is one important effector of this p53/miR-30a pathway. We demonstrate that the reduction in miR-30a expression elicited by p53 inactivation results in an alleviation of miR-30a-mediated targeting of ZEB2, which correlates with increased cell plasticity, migration and in vivo dissemination. Thus, our results of a connection between p53 and EMT via miR-30a, with ZEB2 as a novel actor in this play, indicate that the p53/miR-30a/ZEB2 axis contributes to the poor outcome of TNBC.

Physiologically, the ZEB family of transcription factors play a pivotal role in neural crest formation and migration during embryo development, a process involving EMT [49]. In recent years, ZEB factors have gained attention for their pro-oncogenic functions. As reported for other EMT transcription factors, the expression of ZEB proteins in tumor cells contributes to the shift from an epithelial towards a more mesenchymal phenotype and concurrently confers resistance to DNA damage, apoptosis and premature senescence [32, 50].

Overexpression of ZEB proteins has been shown to contribute to several cancer types [51, 52] and, in particular, it is considered an unfavorable factor in BC [25, 53]. ZEB proteins have been previously linked to p53 via miR-200 [13], a family of p53-regulated miRNAs. In fact, miR-200s establish with ZEB a double-negative feedback loop [28, 32, 33, 54]. Our results indicate that miR-30a complements this equation. In fact, miR-30a and miR-200c levels are positively correlated in human BC and miR-30a-induced downmodulation of ZEB2 results in miR-200c overexpression.

Overall, this study demonstrates the existence of a novel axis, p53/miR-30a/ZEB2, that links p53 inactivation to EMT and BC aggressiveness (Fig. 5c), and adds support to the notion that the control of p53 over the various tumoral phenotypes relies on convergent and integrated circuits, in which miRNAs appear as emerging and pivotal nodes.

## Materials and methods

### Cell models

The human breast cancer cell lines MDA-MB-157, MDA-MB-231, MDA-MB-436 (here referred as MDA157, MDA231 and MDA436), Hs578T, BT549, HCC1395 and HCT116 colorectal cancer cell lines were obtained from the ATCC, and HBL100 from Interlab Cell Line Collection-Genova. All cell lines, periodically authenticated by short tandem repeat profiling and tested mycoplasma-negative, were cultured as previously described [55].

siRNAs for p53 (HSS110905, HSS186390, HSS186391; ThermoFisher Scientific) and non-targeting siRNA (12935-100; ThermoFisher Scientific) were transfected using Lipofectamine 3000 (ThermoFisher Scientific). Ambion Pre-miR miRNA precursors specific either for the 5p or the 3p strand (Life Technologies, ThermoFisher Scientific), anti-miRNAs (anti-miR) and relative controls (Life Technologies, ThermoFisher Scientific) were transfected with the siPORT NeoFX Transfection reagent (ThermoFisher Scientific) according to the manufacturer’s instructions. Two ON-Target-plus siRNAs for ZEB2 (J-006914-22, J-006914-23; Dharmacon) and a non-targeting siRNA were transfected using the DharmaFECT reagent 4 (ThermoFisher Scientific).

Lentiviral delivery, carried out as previously described [33], was used to generate stable cell models. MDA157 cells stably expressing p53 were generated by using the pLenti6/V5-p53_wt p53 vector (Addgene plasmid # 22945 [56]). pLenti6GFP was used as a control. MDA231 cells were engineered to overexpress ZEB2 by transducting the pLJM-ZEB2 lentiviral vector or pLJM1-EGFP (Addgene # 19319 [57]) as a control. To generate pLJM-ZEB2, ZEB2 coding sequence was amplified from TOPO-Blunt-ZEB2 vector (HsCD000347714; Harvard Medical School, Boston) with primers containing* Age*I and *Bst*BI sites and cloned into pLJM1-EGFP.

ZEB2-silenced (sh1-ZEB2, sh2-ZEB2 or sh5-ZEB2) and control cells (shGFP) (MDA231, MDA157, HBL100, Hs578T, BT549 and HCC1395) were generated by using lentiviral plasmids obtained from a modified version of pRSI9 DECIPHER vector (Cellecta) in which an *Age*I site was introduced by mutagenesis. Individual sequences for shZEB2 and shGFP (Supplementary Table S1) were cloned in the *Age*I and *Eco*RI sites.

The *MIR200C* promoter is notoriously constitutively methylated in the TNBC cell lines and several cell divisions are needed to achieve appreciable reactivation by demethylating agents [33, 58]. Thus, stable cell models were needed to address the hypothesis of a miR-30a/ZEB2-mediated activation of miR-200c. To this end, MDA231, MDA157 and HBL100 were engineered to express miR-30a (5p/3p) or silenced for ZEB2 via lentiviral delivery. Constitutive miR-30a (5p/3p) overexpression was achieved by lentiviral infection with pLenti6-miR-30a-(5p/3p) or pLenti6GFP vectors. *MIR30A* (miR-30a (5p/3p)) genomic region, amplified from MCF7 genomic DNA with primers containing *Xho*I and *Not*I sites (Supplementary Table S1), was initially cloned in pLNCX2-vector. The *MIR30A* fragment was then cleaved with *Bgl*II and *Cla*I restriction enzymes and inserted into *Bam*HI and BstBI restriction sites of pLenti6GFP.

### TCGA dataset

A dataset of 249 BC samples comprising clinicopathological information, miRNA-seq data and *TP53* mutational status was retrieved (on December 2014) from TCGA portal (http://tcga-data.nci.nih.gov/tcga/findArchives.htm [16]). The set included 204 BC hormonal receptor positive, 27 TNBC and 13 expressing HER2 but lacking hormonal receptors (5 cases were unknown). Besides, 163 BCs presented wild-type *TP53* and 82 carried *TP53* mutations (4 were unknown). miRNAs data of paired tumor and normal tissues were collected for 12 cases. miRNA-seq data and clinicopathological records of further 63 TNBC were downloaded from TCGA data portal. Collected miRNA-seq data (level 3) included the calculated expression for all reads aligned to a specific miRNA reported as RPM (reads per million miRNA mapped). Comparisons were performed on LOG2 of RPM values. Analyses reported in Table 1 were performed by using data from mirnas.quantification files; in the analyses of miR-30a isoforms the isoform.quantification files were used.

### Patients and samples

Formalin-fixed, paraffin-embedded (FFPE) specimens of 59 TNBCs were retrospectively collected at the CRO Aviano National Cancer Institute biobank (2000–2010). Cases were selected based on the following criteria: naive for chemotherapy and radiotherapy, tumor cellularity greater than 70%, suitability of the material for molecular analyses. Informed consent was obtained and use of patient samples was approved by the Institutional Review Board. Clinicopathological and follow-up data were retrieved from clinical records (Table 2).

### RNA extraction and qRT-PCR

Total RNA was isolated from FFPE tumors samples using the Recover All Total Nucleic Acid Isolation Kit (ThermoFisher Scientific). The miRNeasy Mini Kit (Qiagen) was used to isolate total RNA from cell lines. Complementary DNA was generated by using SuperScriptIII-Reverse Transcriptase (Applied Biosystems, ThermoFisher Scientific) for pri-miRNA detection and by TaqMan MicroRNA Reverse Transcription Kit (Life Technologies, ThermoFisher Scientific) for miRNA analyses. Pri-miR-30a, miRNAs (miR-30a-5p, miR-30a-3p, miR-200c) and RNU48 and RNU6B (reference genes) were then amplified by quantitative reverse transcription-PCR (qRT-PCR) using TaqMan-specific kits (Life Technologies, ThermoFisher Scientific). Relative expression levels were normalized to controls (geometric mean of the reference genes) by using the comparative Ct (ΔΔCt) method and the Bio-Rad CFX manager software. All experiments were done in triplicate and confirmed in at least three independent experiments.

### Dual-Luciferase reporter assay

The miR-30a promoter region was amplified from genomic DNA extracted from MCF7 cells and cloned into the pGL3 basic Luciferase vector (Promega). The 3’UTR of *ZEB2* was amplified from genomic DNA extracted from MDA231 and cloned in pMIR-REPORT^TM^ Luciferase vector (ThermoFisher Scientific). The p53 binding sites identified on the miR-30a promoter by the MatInspector software (Genomatix Software GmbH, Munich, Germany) and the miR-30a-5p and miR-30a-3p binding sites on the 3’UTR of *ZEB2* were modified by site-direct mutagenesis (QuikChangeTM Site-Directed Mutagenesis Kit, Stratagene). The primers used for amplification and mutagenesis are reported in Supplementary Table S1.

Reporter plasmids were transiently transfected in the indicated cell lines using the Lipofectamine 3000 reagent (ThermoFisher Scientific); pCMV-*Renilla* or PGK-*Renilla* were used for normalization. siRNA and pre-miRNA were transfected at 25 nM final concentration. Reporter assays were performed 48 h after transfection using the Dual-luciferase assay system (Promega). Transfection efficiency was normalized by calculating the Luciferase/*Renilla* activity ratio. All experiments were done in triplicate and data confirmed in at least three independent experiments.

### Western blot analyses

Protein extraction and western blot were performed as previously described [55]. Detailed description of the used antibodies is reported in Supplementary Table S2. Immunoreactivity was detected with anti-mouse and anti-rabbit secondary antibodies horseradish peroxidase labeled (PerkinElmer) using Western Lightning^™^ Chemiluminescence Reagent Plus (PerkinElmer). Images were captured and analyzed using the Chemidoc XRS+ system (Bio-Rad). Expression levels were quantified using the ImageLab imaging software (Bio-Rad). Results were confirmed in at least three independent experiments.

### Chromatin immunoprecipitation

Chromatin crosslinking was performed according to the protocol developed by P.J. Farnham (available online at: http://farnham.genomecenter.ucdavis.edu/pdf/FarnhamLabChIP%20Protocol.pdf).

For ChIP, 4 µg of DO-1 anti-p53 monoclonal antibody or isotype-matched pre-immune mouse IgG, as a negative control, were used. Quantitative real-time PCRs with the EvaGreen dye technology (Bio-Rad) was used to quantify the DNA in ChIP samples. Analysis of ChIP data was carried out using the fold enrichment method normalized to mock (IgG) control for each sample (ThermoFisher Scientific, https://tools.thermofisher.com/content/sfs/brochures/Step-by-Step-Guide-to-Successful-ChIP-Assays.pdf). Details about oligonucleotides and antibodies are reported in Supplementary Table S1 and S2, respectively. The oligonucleotides used for positive and negative controls were as previously described [59]. The results were confirmed in two independent experiments.

### Migration assays

Migration assays were performed on several cell models modulated for ZEB2 and/or miR-30a expression, namely: MDA231, MDA157, BT549, Hs578T and HCC1395 stably silenced for ZEB2 via lentiviral delivery; ZEB2-silenced/miR-30a overexpressing MDA231 cell models, generated by first transfecting ZEB2-specific siRNAs or non-targeting siRNA (ThermoFisher Scientific, 25 nM) and then (24 h later) by further transfecting pre-miR-30a-5p, pre-miR-30a-3p or pre-miR control (Ambion, 5 nM); MDA231 cells engineered to stably express ZEB2 or control, transfected with pre-miR-30a-5p, pre-miR-30a-3p or pre-miRNA control (Ambion, 5 nM); MDA231 cells transfected with anti-miRNAs and relative control (ThermoFisher Scientific, 10 nM). At 48 h post transfection, cells were collected for subsequent analyses.

Migration assays were performed as described in Spessotto et al. [60]. Briefly, cells were trypsinized, collected and fluorescently labeled with Fast DiI dye solution (Molecular Probes, Inc.) for 10 min at 37 °C in 5% CO_2_. Cells were then washed in serum-free medium and seeded (10^5^ cells/insert) in serum-free medium on the top side of Fluoroblok inserts (Corning). Medium containing 10% of fetal bovine serum was used as chemoattractant in the lower chamber (bottom side). Fluorescence intensity at 576 nm of top (nonmigrated cells) and bottom (transmigrated cells) side of the well was measured at the indicated time points (*tx*, 7 or 24 h) using a microplate reader (Infinite M1000PRO, TECAN). The percentage of transmigrated cells was determined as follows: 100×(FB_*tx*_−FB_*t*0_)/FT_*t*0_ where FB_tx_ is the fluorescence intensity of bottom side at the indicated time point; FB_*t*0_ is the fluorescence intensity of bottom side at the time zero; FT_*t*0_ is fluorescence intensity of top side at the time zero. All experiments were performed in triplicate and data confirmed in at least three independent experiments.

### Zebrafish in vivo experiments

To address the role of the mir-30a/ZEB2 axis in vivo, experiments were performed using the zebrafish embryo model. We choose this model because, beside meeting the 3R recommendations of using animals with a reduced nervous system development, its transparency allows an effective and real-time assessment of tumor cell growth and migration.

The transgenic zebrafish line Tg (fli1:EGFP), expressing enhanced green fluorescent protein (EGFP) in endothelial cells in wild-type background, was used for in vivo evaluation of tumor cell dissemination and micrometastatization. Zebrafish and embryos were raised, staged and maintained according to standard procedures (http://ZFIN.org) in compliance with the local animal welfare regulations.

BC cells, engineered as specified in the text and made fluorescent following infection with the mCHERRY lentiviral vector (pCMV-mCherry-bc-puro-Kl201), were injected in the yolk sac and analyzed as previously described [29]. Tumor dissemination was measured as “spreading distance per object” representing the mean cell migration for each embryo.

To investigate the ability of engineered cells to extravasate and form distal metastasis, mCHERRY-positive BC cells were injected into the duct of Cuvier of 2-day-old fli1:EGFP embryos. The fraction of embryos exhibiting micrometastastatic colonization of the caudal fin (>10 cells) was calculated at 1, 4 and 6 dpi (days post injection), as previously described [31]. Data are representative of two independent experiments with at least 24 embryos per group. All experiments were performed twice.

### Statistical analyses

For miRNA expression analysis tumor samples were categorized according to the median expression value into “low” (expression levels<median) and “high” (expression levels≥median). The Mann–Whitney–Wilcoxon test and Fisher’s exact test were used to assess associations between miRNA expression and selected prognostic factors (age at diagnosis, tumor size, lymph nodes status, metastasis, TNM (tumor, node, metastasis) stage, tumor grade and treatments). Survival analyses were conducted considering the time from diagnosis to the date of the event (death, relapse or last follow-up). Overall and disease-free survivals were estimated using the Kaplan–Meier method and differences between curves were evaluated using the log-rank test. Differences in miRNA expression levels between groups were assessed by using Mann–Whitney rank sum test for TCGA dataset (values not normally distributed) and by *t*-test for the in-house series (values normally distributed and equal variance between groups). Statistical analyses for in vitro experiments were performed using two-tailed *t*-test. Correlation between miRNA levels was evaluated by calculating Spearman’s correlation coefficient (*r*). Statistical analyses were performed with SAS 9.4 (SAS Institute Inc.) and SigmaPlot (Systat Software Inc.).

## Electronic supplementary material

Supplementary Information
