# Supplementary material for: A p53/miR-30a/ZEB2 axis controls triple negative breast cancer aggressiveness
Source: Cell Death Differ. 2018 Apr 17;25(12):2165–80. doi: 10.1038/s41418-018-0103-x (PMC6262018; doi:10.1038/s41418-018-0103-x)
Supplement: Supplementary file 1 — Supplementary Information [file 41418_2018_103_MOESM1_ESM.pdf]

Supplementary Information

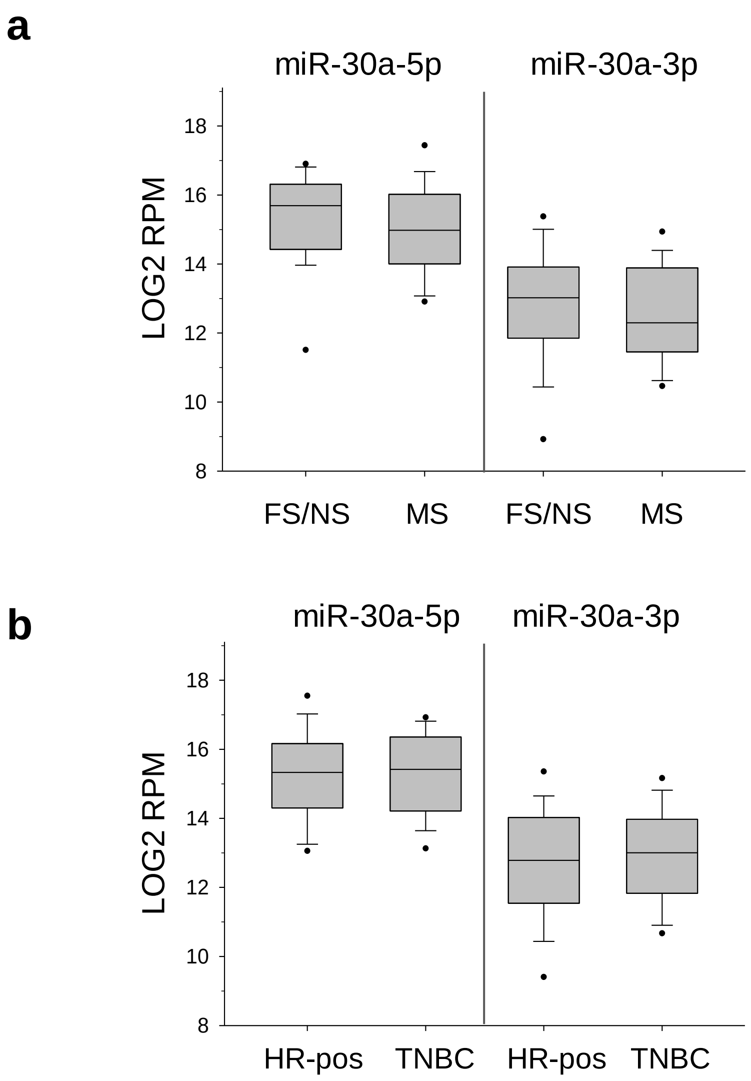

Supplementary Figure S1

miR-30a expression in breast cancers (TCGA series) in relation to the type of *TP53* mutation or molecular subtype.

Data were obtained from TCGA (http://tcga-data.nci.nih.gov/tcga/findArchives.htm). Expression levels are expressed as LOG2 RPM (reads per million miRNA mapped). Groups were compared by using the Mann-Whitney Rank Sum Test.
Lines within the boxes mark the median, boundaries of the boxes represent the 25th percentile, and the 75th percentile, whiskers below and above the boxes indicate the 5th and 95th percentiles.
a) The expression levels of miR-30a-5p and miR-30a-3p did not significantly differ between tumors carrying frameshift/nonsense mutations (FS/NS, 29 cases) *vs* missense mutations (MS, 53 cases) (p=0.179 for miR-30a-5p; p= 0.325 for miR-30a-3p).
b) miR-30a-5p and miR-30a-3p levels are similar in hormone receptor-positive (45 cases, HR pos) and in triple negative (24 cases, TNBC) *TP53* mutated tumors (p= 0.96 for miR-30a-5p ; p=0.77 for miR-30a-3p .

a

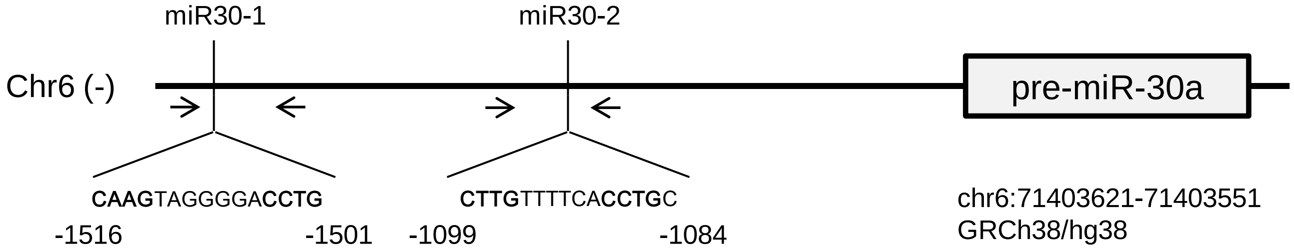

b

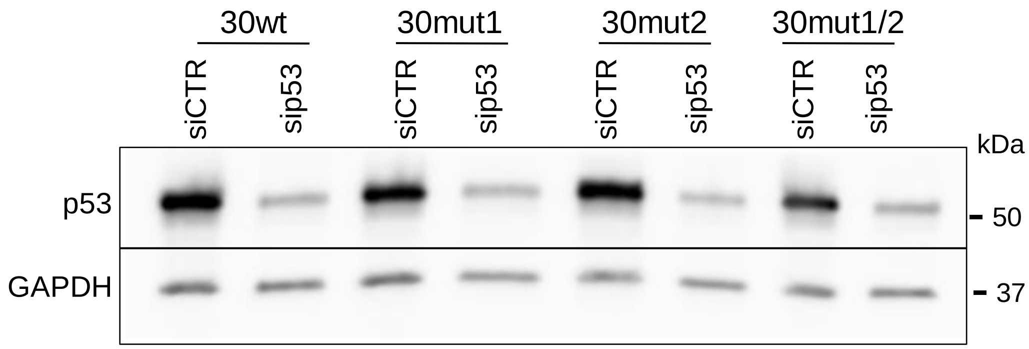

Supplementary Figure S2

a) Schematic representation of the genomic region upstream the pre-miR-30a locus and encompassing the putative promoter region of *MIR30a* (chr6, reverse-complementary strand in hg38).
The two p53 binding sites (miR-30a-1 and miR-30a-2; MatInspector matrix similarity score >0 .8) are indicated.
Consensus sequences are reported in bold.
Arrows indicate the position of the primers used in ChIP experiments (sequences provided in Supplementary Table S1).
b) Western blot showing p53 expression in HCT116 cells transfected with *MIR30a*-luciferase reporter together with either control siRNA (siCTR) or a siRNA targeting p53 (sip53).

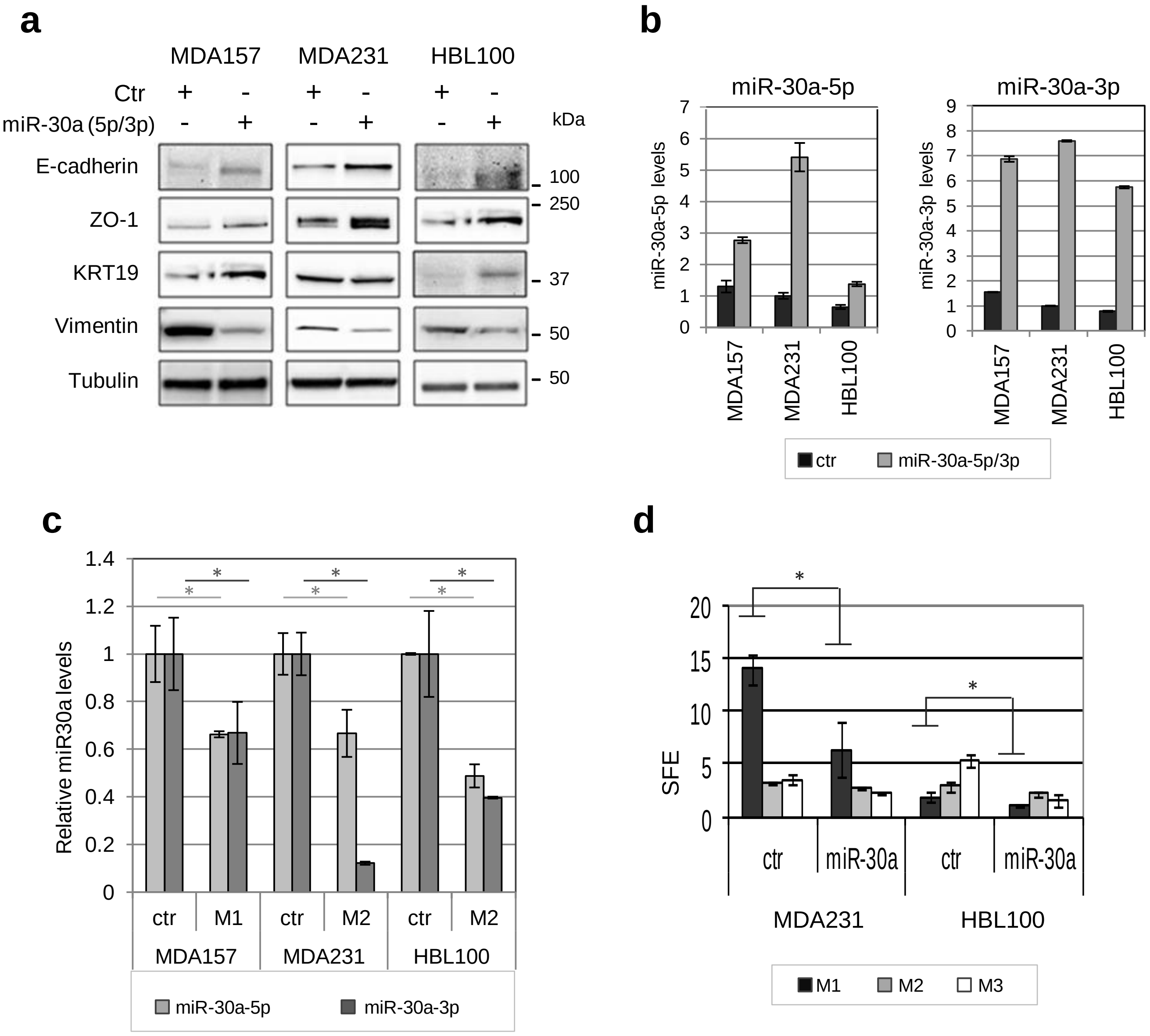

Supplementary Figure S3

miR-30a affects EMT and cell plasticity

a) Immunoblots showing the increment of epithelial proteins E-cadherin, ZO-1, KRT19 and the decrease of the mesenchymal marker Vimentin in MDA157, MDA231 and HBL100 cell lines stably over-expressing miR30a (5p/3p). ctr, control cells transduced with empty vector.
b) miR-30a-5p and miR-30a-3p levels in the cell models described in (a), measured by qRT-PCR.
c) Relative expression levels of miR-30a-5p and miR-30a-3p, measured by qRT-PCR, in MDA157, MDA231 and HBL100 cells grown as mammospheres.
d) Sphere forming efficiency (SFE) of the cell models described in (a). M1, M2 and M3 indicate primary, secondary and tertiary mammospheres, respectively.
Data represent the mean of three independent experiments ± SD; * $p<0.05$.

a

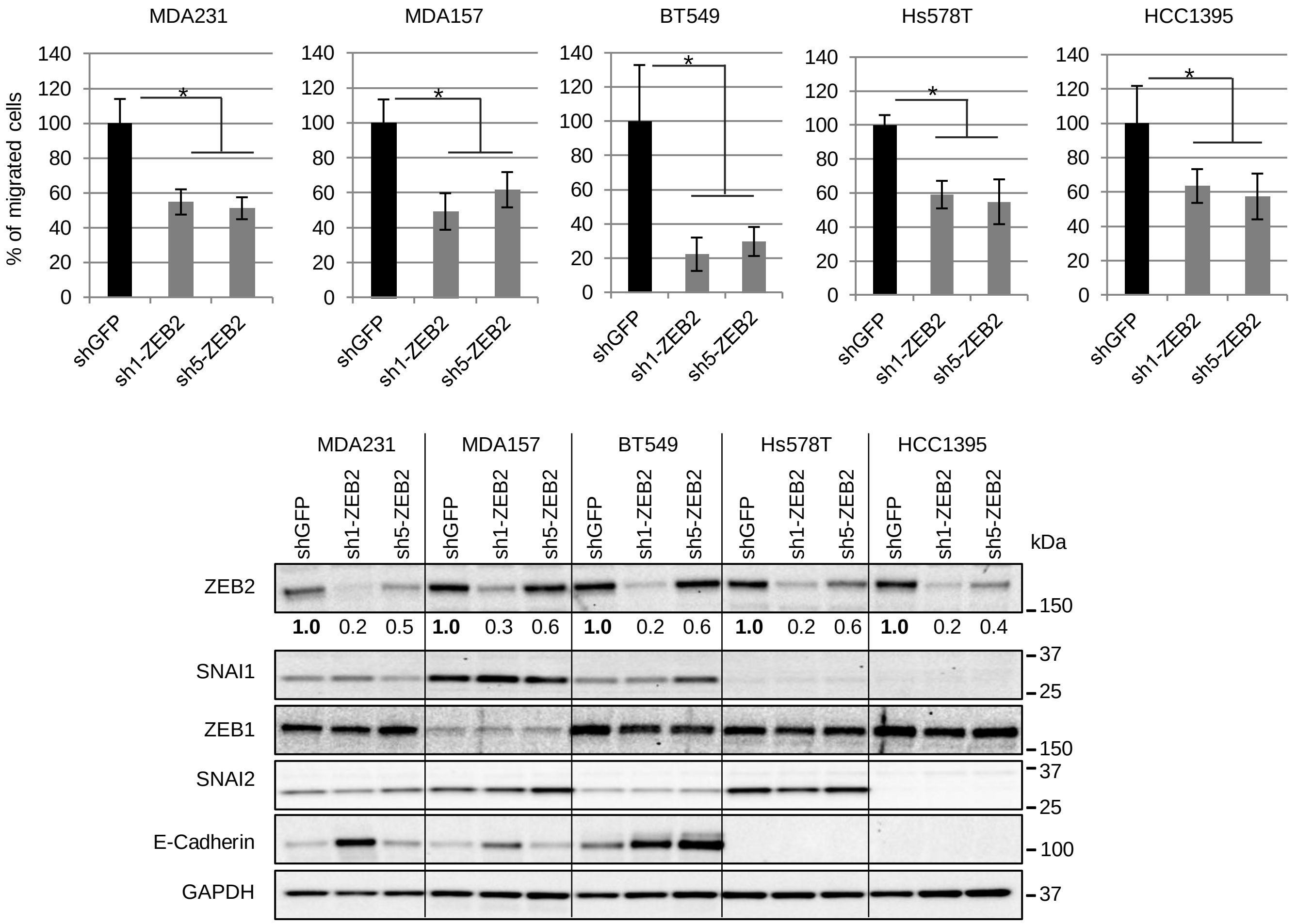

b

	Nr of cases tested	Nr of cases showing ZEB2 reactivity in tumor cells (%)
Luminal A	9	0 (00.0)
Luminal B	16	4 (25.0)
HER2	7	2 (28.6)
Basal-Like/TNBC	19	9 (47.4)

Fisher exact test , p=0.066

c

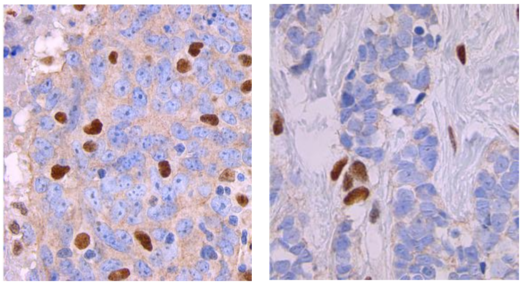

Supplementary Figure S4

Role of ZEB2 in breast cancers

a) Upper panel: Effect of ZEB2 downregulation (sh1-ZEB2 and sh5-ZEB2) on the migration capability of a panel of TNBC cell lines. Percentages of migrated cells were measured at 7h for MDA231 and Hs578T; at 24h for MDA157, BT549 and HCC1395. Data represent the mean of two independent experiments ± SD; * $p<0.05$. Lower panel: Immunoblots showing the expression of ZEB2, SNAI1, ZEB1, SNAI2 and E-cadherin in the same cell models. Numbers under the blot indicate ZEB2 relative levels normalized over GAPDH (loading control). shGFP control was set to 1.
b) ZEB2 expression in the different BC subtypes as assessed by immunohistochemistry (antibody HPA003456 anti-ZEB2, Sigma Aldrich).
c) Representative images of ZEB2 immunoreactivity in two basal-like/triple negative breast cancers.

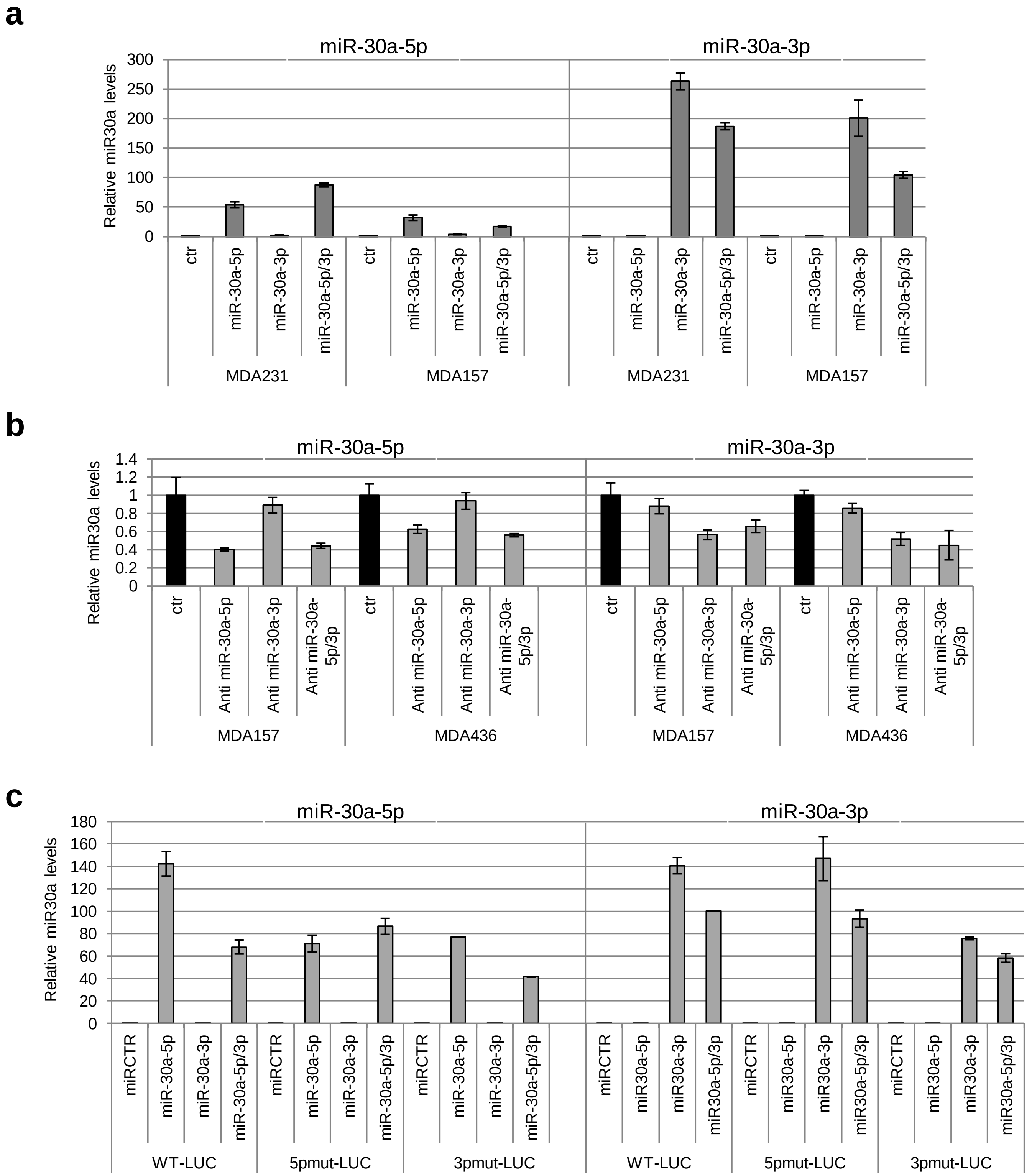

Supplementary Figure S5

Characterization of the cell models used in Figure 3

a) Relative expression of miR-30a-5p and miR-30a-3p in MDA231 and MDA157 transiently transfected with a control miRNA (ctr), miR-30a-5p, miR-30a-3p or a combination of the two miRNAs (miR30a-5p/3p).
b) Expression levels of miR-30a-5p and miR-30a-3p in cells transiently transfected with a control Anti-miRNA (ctr) or Anti-miRNAs directed against miR-30a-5p, miR-30a-3p, alone or in combination (Anti miR-30a-5p/3p).
c) Expression levels of miR-30a-5p and miR-30a-3p in MDA231 cells transfected with the indicated luciferase reporter together a control miRNA (miRCTR), miR-30a-5p or miR-30a-3p, alone or in combination (miR30a-5p/3p).
Bars represent SD

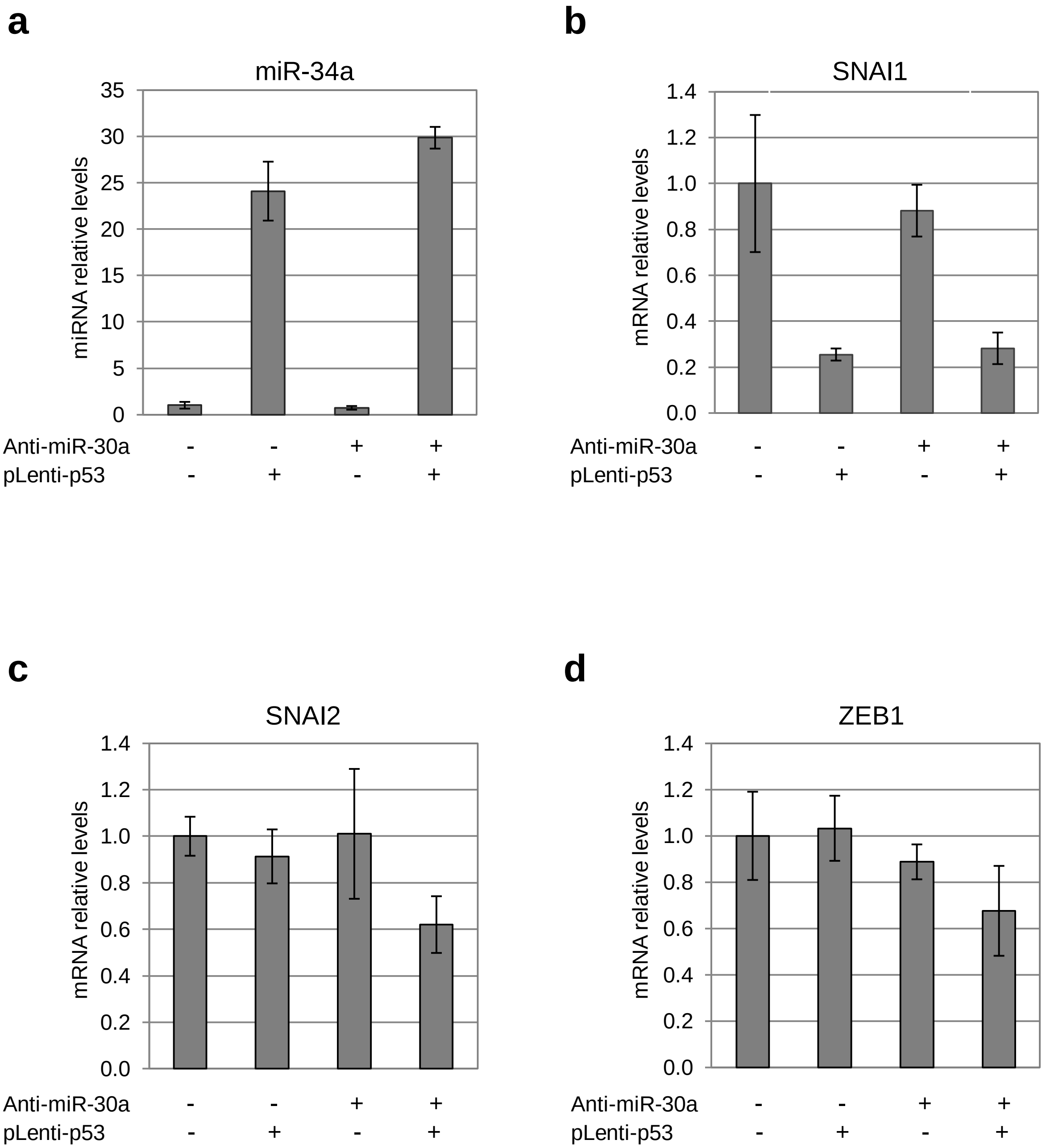

Supplementary Figure S6

Characterization of MDA157 cells overexpressing p53 (pLENTI-p53) and transfected with Anti-miR-30a-5p and -3p

Relative expression levels of miR-34a **(a)**, SNAI1 **(b)**, SNAI2 **(c)** and ZEB1 **(d)** were determined by qRT-PCR.
The expression of miR-34 was quantified with a TaqMan kit (Life Technologies, ThermoFisher Scientific).
A TaqMan assay was set up to measure SNAI1 levels (SNAI1 sense primer cccacctccggagatcctca ; SNAI1 antisense primer ggacagagtcccagatgagc; SNAI1 probe [6FAM]gcgagctgcaggactctaat).
SNAI2 and ZEB1 mRNA levels were measured by qRT-PCR as previously described (55).
Bars represent SD.

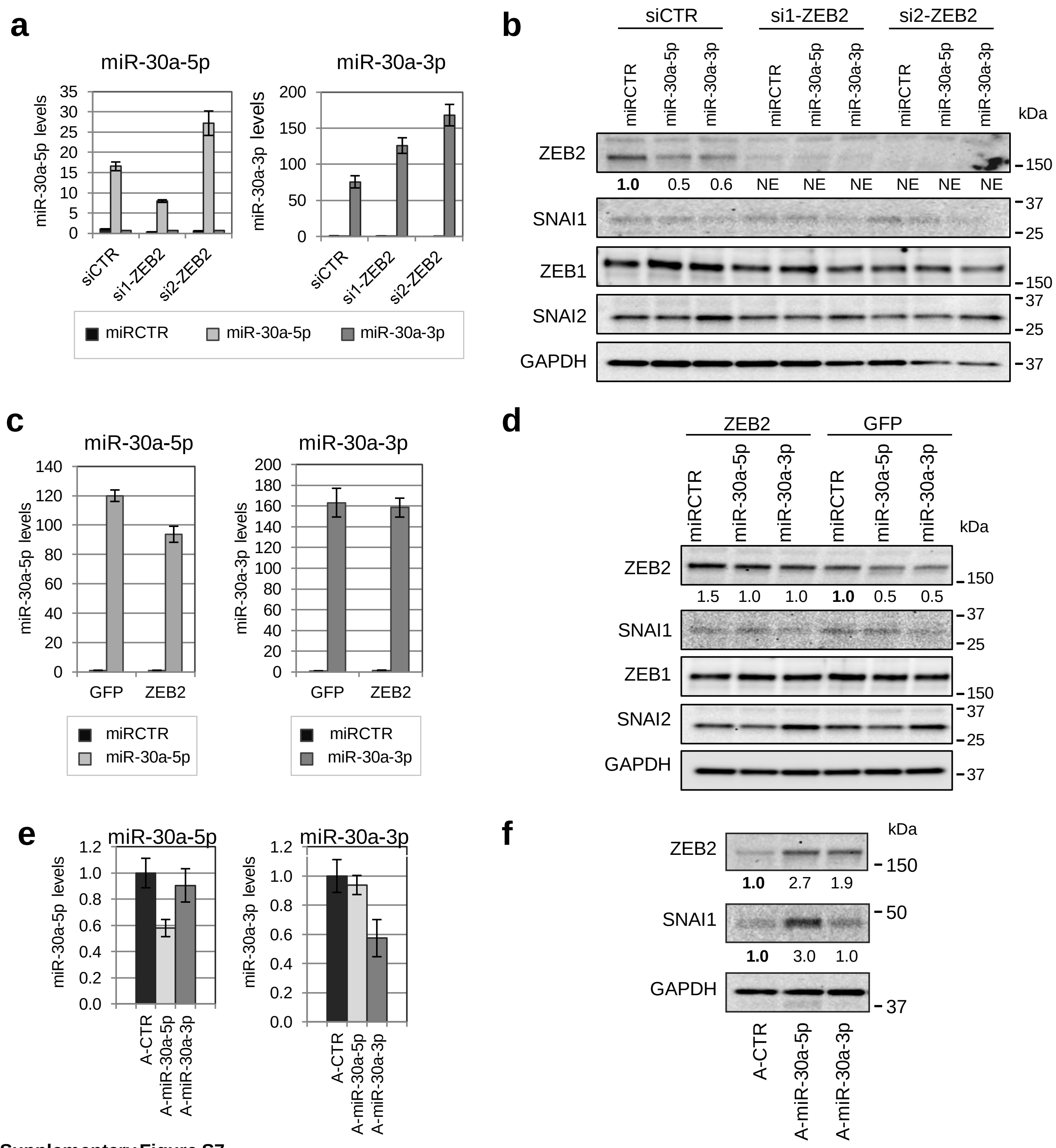

Supplementary Figure S7

Characterization of MDA231 cell models used in figure 4a and 4b

a-b) MDA231 cells silenced for ZEB2 (two different siRNAs, si1-ZEB2 and si2-ZEB2) and engineered to express miR-30a-5p, miR-30a-3p or miRNA negative control were analyzed for miR-30a-5p and -3p expression (a) and for ZEB2, ZEB1, SNAI1 and SNAI2 protein levels (b).
c-d) MDA231 overexpressing ZEB2 or GFP control vector and transfected with miR-30a-5p or miR-30a-3p or miRNA negative control were analyzed for miR-30a-5p and -3p expression (c) and for ZEB2, ZEB1, SNAI1 and SNAI2 protein levels (d).
e-f) MDA231 transfected with Anti-miR-30a (A-miR-30a-5p and A-miR-30a-3p) or Anti-miR-negative control (A-CTR) were analyzed for miR-30a-5p and -3p expression (e) and ZEB2 and SNAI1 protein levels (f).
miRNA levels were measured by qRT-PCR (bars represent SD). Protein levels were determined by immunoblot. The numbers below the blots in (b), (d) and (f) indicate ZEB2 relative expression normalized over GAPDH (loading control). NE, not evaluable. Control cells (miRCTR-siCTR, miRCTR-GFP and A-CTR, respectively) were set to 1.

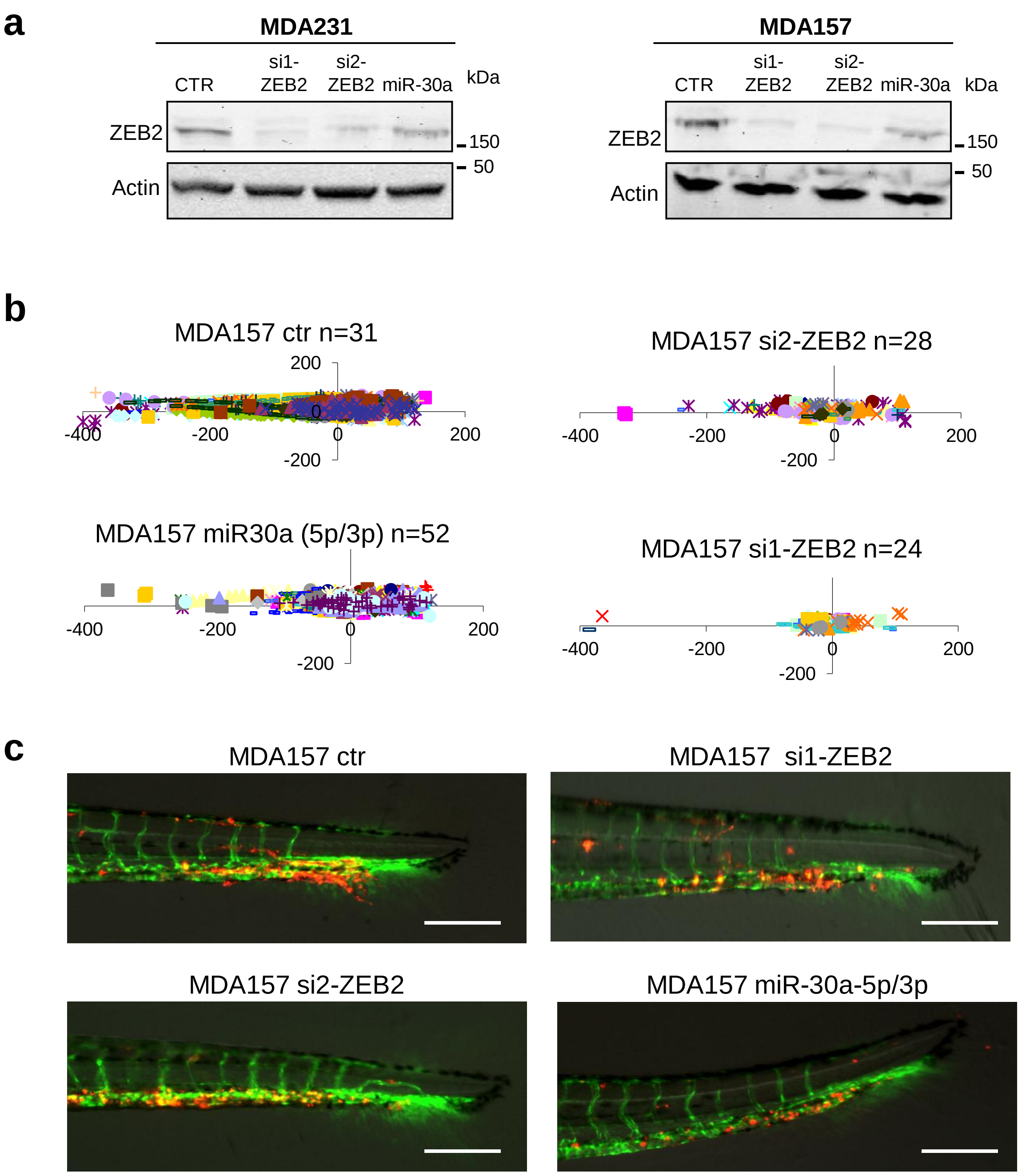

Supplementary Figure S8

Spreading and micrometastasis formation in zebrafish embryos by MDA157 cells silenced for ZEB2 or engineered to express miR-30a (5p/3p)

a) Immunoblots showing the ZEB2 expression in the cell models used to investigate cells spreading in zebrafish embryos. mCHERRY MDA231 and mCHERRY MDA157 cells were silenced for ZEB2 (two different siRNAs, si1-ZEB2 and si2-ZEB2) or engineered to express miR-30a-5p and -3p. Cells transfected with an empty vector were used as a control (ctr). Actin (Act) was used as a loading control.
b) Scatter plot representing the dissemination in zebrafish embryos of the MDA157 models described above. Cells were implanted in the yolk sac of 2 days-old embryos (fli1:EGFP strain). Embryos were automatically imaged at 6dpi. Dots represent single cells; colors identify each microinjected embryo; x axis indicate the migration from the injection point (0,0) toward the head (positive values) or the tail (negative values); n indicates the number of embryos analyzed.
c) Representative images of zebrafishes injected with the indicated MDA157 cell models at 6 dpi. Cells were injected into the blood circulation of 2 days-old zebrafish embryos. Scale bar = 100 µm.

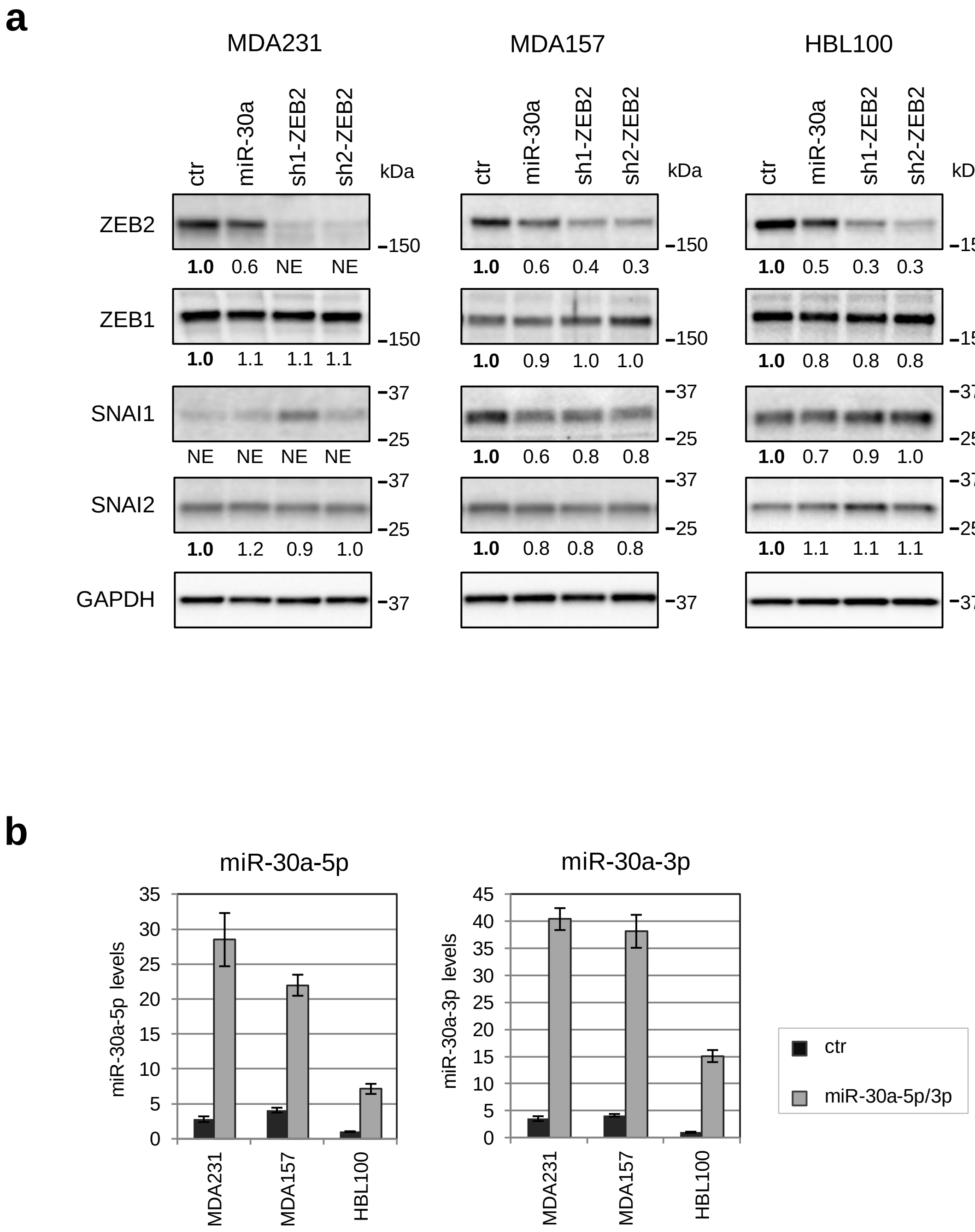

Supplementary Figure S9

Characterization of MDA231, MDA157 and HBL100 cell models used in Figure 5a

Cells were engineered to ectopically express miR-30a-5p/3p (miR-30a) or were depleted for ZEB2 (sh1-ZEB2 and sh2-ZEB2). Control vectors were pLenti6GFP and shGFP, respectively.
a) ZEB2, ZEB1, SNA1 and SNAI2 protein levels as assessed by immunoblots. The numbers below the blots indicate the relative expression levels normalized over GAPDH (loading control). Ctr, here set to 1, represents the expression levels of pLenti6GFP-infected cells. However, the two control vectors (pLenti6GFP and shGFP) yielded similar results. NE, not evaluable.
(b) miR-30a-5p and miR-30a-3p expression prior (black) and after (grey) miR30a delivery measured by qRT-PCR (bars represent SD).

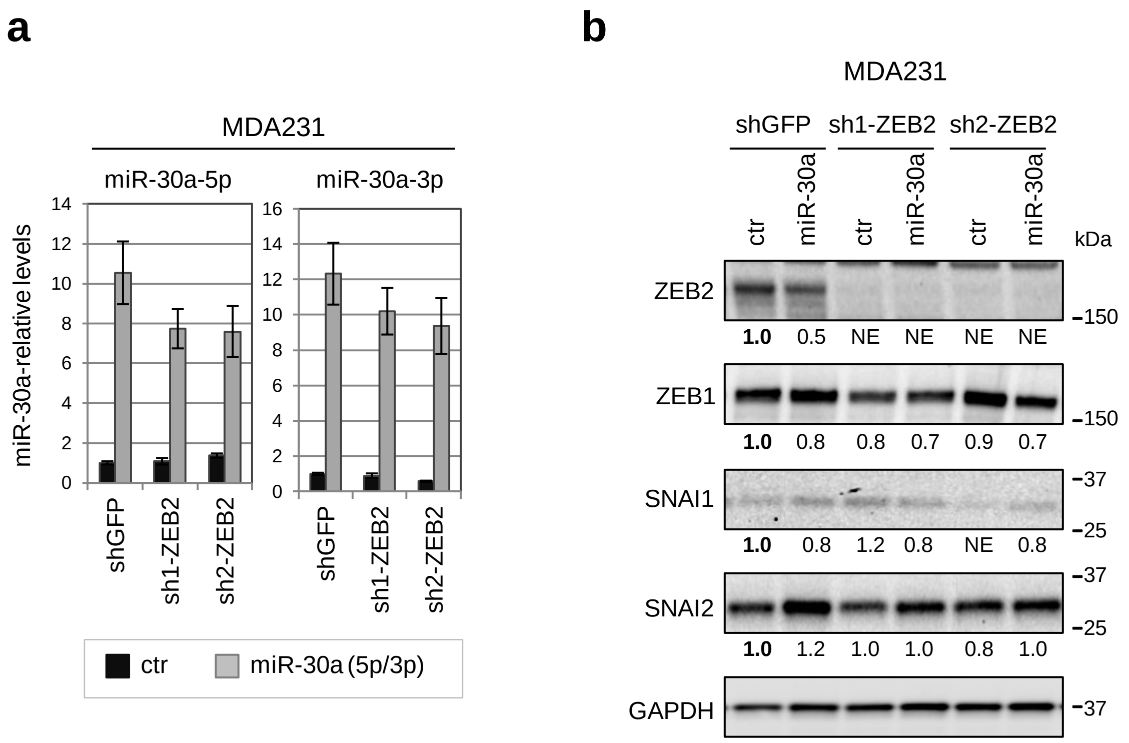

Supplementary Figure S10

Characterization of the cell models used in Figure 5b

ZEB2 and miR-30a-5p and miR-30a-3p expression in MDA231 cells proficient (shGFP) or deficient for ZEB2 (sh1-ZEB2 and sh2-ZEB2) further engineered to ectopically express miR-30a-5p/3p (miR-30a) or pLenti6GFP control vector (ctr).
a) qRT-PCR showing miR-30a-5p and miR-30a-3p relative levels (bars represent SD).
b) Immunoblots showing the expression of ZEB2, ZEB1, SNAI1 and SNAI2. The numbers below the blots indicate the relative expression levels normalized over the GAPDH blot (loading control). shGFP-ctr was set to 1. NE, not evaluable.

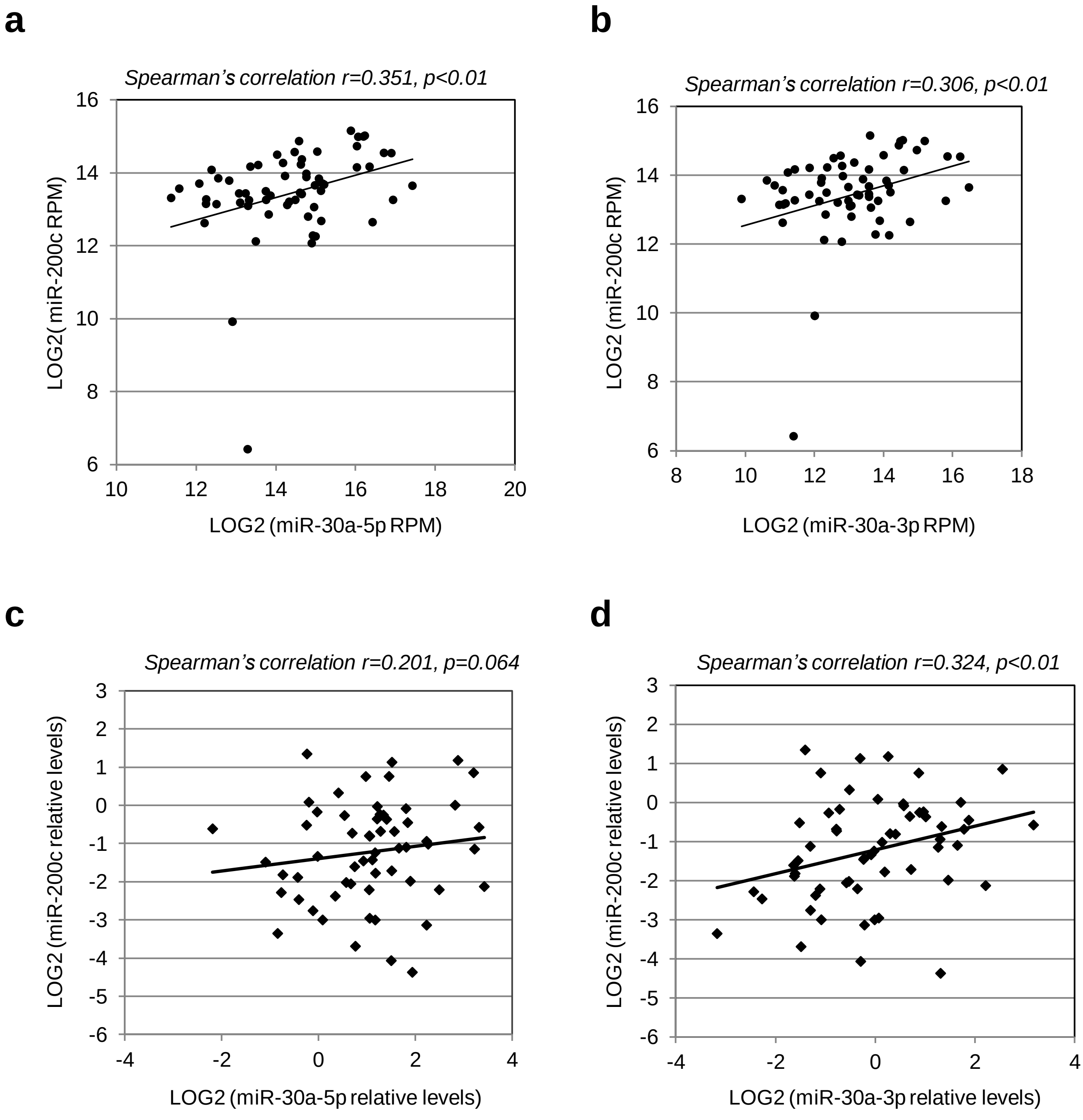

Supplementary Figure S11

miR-30a correlates with miR-200c

miR-30a levels positively correlate with miR-200c in the TCGA TNBC series (63 cases)
a) Scatter plot showing the correlation between miR-200c and miR-30a-5p and
b) between miR-200c and miR-30a-3p.
Data are reported as LOG2 RPM values (RPM, reads per million miRNAs mapped)

miR-30a levels positively correlate with miR-200c in the in house TNBC series (59 cases)
c) Scatter plot showing the correlation between miR-200c and miR-30a-5p and
d) between miR-200c andmiR-30a-3p .
Data are reported as LOG2 relative miRNA levels (assessed by qRT-PCR).

Supplementary Table S1

Primer sequences for DNA constructs

Oligo	Sense primer	Antisense primer
pLNCX2-miR-30a-5p/3p	CCGCTCGAGCTAGAAGCTCGGTGATGAATAATAGACATC	ATAAGAATGCGGCCGC TGCATACACAGAGCACCTCCTCAAT
shGFP	CCGGCAACAGCCACAACGTCTATGTTAATATTCATAGCATAGACGTTGTGGCTGTTGTTTTTT	AATTAAAAAACAACAGCCACAACGTCTATGCTATGAATATTAACATAGACGTTGTGGCTGTTG
sh1-ZEB2	CCGGGCAGTTCCTTAGTTTACATATGTTAATATTCATAGCATATGTAAACTAAGGAACTGCTTTTTT	AATTAAAAAAGCAGTTCCTTAGTTTACATATGCTATGAATATTAACATATGTAAACTAAGGAACTGC
sh2-ZEB2	CCGGCCCGAAATGATATGAGATGAAGTTAATATTCATAGCTTCATCTCGTATCGTTTCGGGTTTTTT	AATTAAAAAACCCGAAACGATACGAGATGAAGCTATGAATATTAACTTCATCTCATATCATTTCGGG
sh5-ZEB2	CCGGCCCACCATGAATAGTAATTTAGTTAATATTCATAGCTAAATTACTATTCATGGTGGGTTTTTT	AATTAAAAAACCCACCATGAATAGTAATTTAGCTATGAATATTAACTAAATTACTATTCATGGTGGG
WT-LUC	GGACTAGTCCTGCTTGAAAACACTGCTG	CCCAAGCTTCGAGCATGGTCATTTTCAAA
5pMUT-LUC	GAATAGGCAGCAGTTCCTTATGGGCACTATGTTTGTGCAATTATTTTC	GAAATAATTGCACAAACATAGTGCCCATAAGGAACTGCTGCCTAATTC
3pMUT-LUC	CTTGTGAAAACACTGTATTTGATCAGTCCCATTCCACTTTCTTCATCTTG	CAAGATGAAGAAAGTGGAATGGGACTGATCAAATACAGTGTTTTCACAAG
30-LUC	CTAGCTAGCCAAAGTGAGAGGGTGGCAAT	GGAAGATCTTCACTGTCAACAGCAATATAC
30mut1-LUC	AAAAGTGGTAAGATTCCAAGGTAATTAGGGGATCTTTCACTTTGAGAAAAATTTATGG	CCATAAATTTTTCTCAAAGTGAAAGATCCCCTAATTACCTTGGAATCTTACCACTTTT
30mut2-LUC	GGCCACAACATAATTCAGTCCTTTTTCATCCTCTGACTCAAAGATTAAC	GTTAATCTTTGAGTCAGAGGATGAAAAAGGACTGAATTATGTTGTGGCC
pLJM-ZEB2	AATCGAACCGGTCGCCACCATGAAGCAGCCGATCATGG	ATCGATTCGAATTACATGCCATCTTCCATATT

Primer sequences for ChIp

Gene symbol	Sense primer	Antisense primer
miR30-1	TCTCACCAAGATACCCACTTCT	GTGGTAAGATTCCAAGGCAAGT
miR30-2	TTTCTCAAAGCAAAGGGTAACTC	GCACAGCACTGATAAAGTTTGC

Supplementary Table S2

Antibodies used in the western blot analyses		
**Protein**	**Clone**	**Company**
ZEB2	A302-474A	Bethyl Laboratories
p53	DO-1 SC-126	Santa Cruz
ZEB1	H-102 SC-25388	Santa Cruz
SNAI1	C15D3 #3879	Cell Signaling Technology
SNAI2	C19G7 #9585	Cell Signaling Technology
Control IgG	SC2025	Santa Cruz
GAPDH	6C5 SC32233	Santa Cruz
Tubulin	T6557	Sigma-Aldrich
Vinculin	H-10 SC25336	Santa Cruz
